# A Boolean Model of the Proliferative Role of the lncRNA XIST in Non-Small Cell Lung Cancer Cells

**DOI:** 10.3390/biology11040480

**Published:** 2022-03-22

**Authors:** Shantanu Gupta, Daner A. Silveira, Ronaldo F. Hashimoto, Jose Carlos M. Mombach

**Affiliations:** 1 Departamento de Ciência da Computação, Instituto de Matemática e Estatística, Universidade de São Paulo, Rua do Matão 1010, São Paulo 05508-090, SP, Brazil; ronaldo@ime.usp.br; 2 Departamento de Física, Universidade Federal de Santa Maria, Santa Maria 97105-900, RS, Brazil; daner.silveira@gmail.com

**Keywords:** lncRNA-XIST, miR-34a, miR-449a, miR-16, feedback loops, NSCLC

## Abstract

**Simple Summary:**

The lncRNA XIST has been verified as an oncogenic gene in non-small cell lung cancer (NSCLC), whereas the tumor suppressors miR-34a, miR-449a, and miR-16 were downregulated in NSCLC. The lncRNA XIST is known to bind to the miR-449a and miR-34a sponges, however, the biological functions linking all these non-coding RNAs (ncRNAs) are still unknown in NSCLC. This study aims to perform dynamic analysis of the gene regulation between these two ncRNAs in NSCLC. Thus, we presented a Boolean model of the plausible network connecting the lncRNA XIST/miR-34a/miR-449a and miR-16 in NSCLC. We observed that miR-449a/miR-34a regulates miR-16 and p21 expression by targeting HDAC1, c-Myc, and the lncRNA XIST. Our results demonstrate that the lncRNA XIST is an attractive target of drug development in NSCLC and that favorable outcomes can be achieved through tumor suppressor miRNAs.

**Abstract:**

The long non-coding RNA X inactivate-specific transcript (lncRNA XIST) has been verified as an oncogenic gene in non-small cell lung cancer (NSCLC) whose regulatory role is largely unknown. The important tumor suppressors, microRNAs: miR-449a and miR-16 are regulated by lncRNA XIST in NSCLC, these miRNAs share numerous common targets and experimental evidence suggests that they synergistically regulate the cell-fate regulation of NSCLC. LncRNA XIST is known to sponge miR-449a and miR-34a, however, the regulatory network connecting all these non-coding RNAs is still unknown. Here we propose a Boolean regulatory network for the G1/S cell cycle checkpoint in NSCLC contemplating the involvement of these non-coding RNAs. Model verification was conducted by comparison with experimental knowledge from NSCLC showing good agreement. The results suggest that miR-449a regulates miR-16 and p21 activity by targeting HDAC1, c-Myc, and the lncRNA XIST. Furthermore, our circuit perturbation simulations show that five circuits are involved in cell fate determination between senescence and apoptosis. The model thus allows pinpointing the direct cell fate mechanisms of NSCLC. Therefore, our results support that lncRNA XIST is an attractive target of drug development in tumor growth and aggressive proliferation of NSCLC, and promising results can be achieved through tumor suppressor miRNAs.

## 1. Introduction

Long non-coding RNAs (lncRNAs), are a type of non-coding RNAs with lengths larger than 200 nucleotides (nt), they are a novel category of controllers in molecular biology [1]. Rising evidence points that lncRNAs are essential players in cancer biology [2]. Moreover, lncRNAs could be also viewed as diagnostic or prognostic markers depending on their impact on clinical characteristics of tumor outcomes [1,2,3]. Nevertheless, the biological mechanisms and clinical importance of lncRNAs in the initiation, development, and progression of NSCLC are remaining mostly unknown. In this context, the long non-coding RNA X-inactive specific transcript (lncRNA XIST) has been studied in a variety of cancers such as gastric cancer [4], glioma [5] including NSCLC [6]. Recent studies have confirmed that lncRNA XIST acts as an oncogene accelerating tumor growth in NSCLC. Tian et al. [7] observed that overexpression of lncRNA XIST is correlated with cisplatin resistance through the targeting miR-144-3p in A549 and H460 NSCLC cell lines. In addition, these authors further demonstrated that knockdown (KO) of lncRNA XIST triggers activation of miR-144-3p expression, which inhibits the proliferation and migration by the induction of apoptosis in NSCLC [7]. MicroRNAs (miRNAs) are class of small non-coding RNAs, with approximately 20 to 25 in length, which regulate gene expression post-transcriptionally [8].

Another evidence comes through Li et al. [9], in brief, Li et al. investigated that overexpression of lncRNA XIST is linked with Transforming growth factor beta (TGF-β)-induced epithelial-mesenchymal transition (EMT) in A549 and H226 cell lines [9]. Moreover, Li and colleagues observed that lncRNA XIST induces tumor metastasis and by Zinc finger E-box-binding homeobox 2 (ZEB2) in NSCLC [9]. Furthermore, Li et al. found that miR-367 and miR-141 are transcriptional target of lncRNA XIST [9]. In addition, these authors demonstrated that ZEB2 is a direct target of miR-367 and miR-141 [9]. Interestingly, Li and colleagues uncovered that upregulation of miR-367 and miR-141 or downregulation of lncRNA XIST inhibit TGF-β-induced EMT and cell migration and invasion in NSCLC cells [9]. Likewise, Wang et al. [10] observed that upregulation of lncRNA XIST is connected to Transforming growth factor beta-1 (TGF-β1)-induced EMT in A549 and H1299 cell lines [10]. They also demonstrated that lncRNA XIST directly targets miR-137 that induces Neurogenic locus notch homolog protein 1 (NOTCH1) expression. Indeed, lncRNA XIST sponges miR-137 and NOTCH1 is directly inhibited by [10]. Wang et al. found that loss-of-function (LoF) of lncRNA XIST enhanced miR-137 expression that inhibits TGF-β1-induced EMT by knockdown (KO) of NOTCH1 in NSCLC [10]. In addition, the knockdown of lncRNA XIST or overexpression of miR-137 repressed tumor growth and TGF-β1-induced EMT in A549 and H1299 cells [10]. Also, lncRNA XIST activates the Wnt/β-catenin signalling pathway through the upregulation of E3 ubiquitin-protein ligase RING1 (RING1) expression by the sponging miR-744 that regulates aggressive proliferation in NSCLC cell lines such as A549, H1299, H23, H522, H460, H1650, and 95D [11]. Finally, the knockdown of lncRNA XIST or upregulation of miR-744 suppresses tumor growth and metastasis in NSCLC [11].

**Figure 1:**
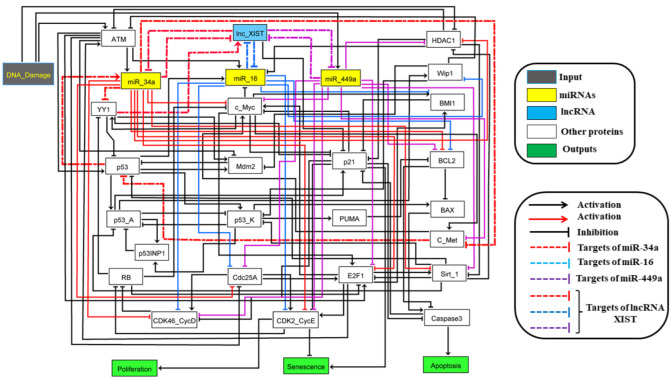
**Boolean model for the regulation of the G1/S checkpoint in response to DNA damage in NSCLC.** miR-34a, miR-16, miR-449a in yellow rectangle, while lncRNA XIST is in the blue rectangle. Dark edges denote activations and red hammerhead edges denote the inhibition by miR-34a of its targets. Blue hammerhead edges denote the inhibition by miR-16 of its targets and violet hammerhead edgesdenote the inhibition by miR-449a. MiR-34a and miR-449a, directly target c-Myc, HDAC1, Sirt-1, E2F1, Cdc25A, BCL2, CDK4/6-cyclin D1 complex (cdk4,6-CycD) and CDK2-cyclin E2 (cdk2-CycE) complex. Whereas, miR-16 targets CDK4,6-CycD, CDK2-cyclin E2, Cdc25A, BCL2, Wip1 and BMI1. For more information about miR-34a and miR-449a targets, see the review by [18,19] and for miR-16 targets, see the reviewed by Aqeilan et al. [20]. The model contains 28 nodes representing proteins and non-coding RNAs and 107 direct interactions among them.

The in vitro experiments mentioned above [7,9,10,11] verify that lncRNA XIST wields its biological consequences on NSCLC typically via interactions with distinct miRNAs. The miR-449a and miR-16 are noteworthy tumor suppressors and play a pivotal role in NSCLC [12,13]. Interestingly, both miRNAs are under-expressed in NSCLC. On the other hand, lncRNA XIST is overexpressed in the same cell line. Recently, the study of Zhang et al. [14] and Zhou et al. [15] evidenced the oncogenic functions of lncRNA XIST by sponging of miR-449a and miR-16 in NSCLC. Similarly, miR-34a is also under-expressed in NSCLC and recently it was observed that miR-34a is is directly inhibited by lncRNA XIST in thyroid cancer [16]. Thus, the study of Zhang et al. [14] and Zhou et al. [15] suggested us to investigate the molecular mechanisms of the axis miR-449a, miR-16, miR-34a and lncRNA XIST in NSCLC. Recently, we have shown that miR-34a can control miR-16 as well as p21 via targeting of HDAC1 and c-Myc in NSCLC and cutaneous T-cell lymphoma (CTCL) [17]. In the present study, we extend our previous model to show how the non-coding RNAs: lncRNA XIST, miR-449a, miR-16, and miR-34a are involved in cell fate regulation induced by DNA damage (Figure 1).

## 2. Materials and Methods

### 2.1. The Molecular Mechanisms of the G1/S Checkpoint in NSCLC

The model includes a single input, DNA damage (Figure 1). The rectangular blue node represents lncRNA XIST, the yellow rectangular nodes represent miR-34a, miR-449a and miR-16 and white rectangular nodes represent proteins. The other in green describe model outputs (Proliferation, Senescence and Apoptosis). Arrows represents activation and hammerhead connectors inhibitions. MiR-34a and miR-449a directly target c-Myc (Figure 1), HDAC1, Sirt-1, E2F1, Cdc25A, BCL2, CDK4/6-cyclin D1 complex (cdk46-CycD) and CDK2/cyclin E2 (cdk2-CycE). Whereas, miR-16 targets CDK4,6/CycD, CDK2/cyclin E2, Cdc25A, BCL2, Wip1 and BMI1, for more information about miR-34a and miR-449a targets, [18,19]. For miR-16 targets, see the reviewed by Aqeilan et al. [20]. The model contains 28 nodes representing proteins and non-coding RNAs and 107 direct interactions among them.

As we mentioned above, the present study is based on our previous study [17]. Here we added a new layer of complexity that we explain below. The presented logical rules that govern the nodes in our model are suggested based on the biochemical literature demonstrated in Table S1.

DNA damage can activated miR-449a expression as observed by Mao et al. [21]. In this Boolean model, we added miR-449a, lncRNA XIST, YY1 and c-Met. The transcription factor Yin-Yang 1 (YY1) is overexpressed in NSCLC and directly activates lncRNA XIST [22]. YY1 directly inhibits miR-34a expression [23] and miR-449a [24]. YY1 is also a negative regulator of p53 activity [25]. Similarly, c-Met is a partner of the sub-family of receptors tyrosine kinases (RTKs) for hepatocyte growth factor (HGF), which is activated by the Myc proto-oncogene protein (c-Myc) and directly inhibits p53 expression [26], while miR-34a and miR-449a inhibit c-Met. In this way, miR-34a and miR-449a can enhanced p53 expression via knockdown YY1 and/or c-Met [27]. Once p53 is triggered in tumor cells in DNA damage response, it can induce p21 for cell cycle arrest for repair and/or senescence or it can induce PUMA and/or BAX for apoptosis. For more details about multistability predicted by our models [17].

### 2.2. Boolean Methods

The structure of the Boolean model of a regulatory network is predicated on the published biochemical knowledge associated with proteins, miRNAs, and lncRNA and their interactions (activatory or inhibitory), which are described by a directed graph. The variable’s values are discrete taking 0 or 1 values. The logical functions are framed supported by the Boolean operators AND, OR, and NOT define the worth of individual nodes in keeping with the influence of its regulators. The Boolean model dynamics in its state-space may be described by a state transition graph, whose nodes define states of the system and whose edges characterize transitions amid these states. The state transition graph serves all the potential trajectories that a single initial state can drive to an ultimate state or attractor. Attractors that haven’t any unreserved edges are named steady states or fixed points, while a collection of transitions trapped in an exceedingly fixed grouping of states form a cyclic state.The importance of the nodes within the system will be revised asynchronously or synchronously. Within the asynchronous scenario, nodes are picked randomly so revised, whereas within the synchronous scenario all nodes are updated at equivalent times. During this work, we only operated the asynchronous update strategy for realism because of its ability to provide non-deterministic modes [28]. Furthermore, the dynamics of a regulatory network are manipulated by negative and positive circuits (also referred to as feedback loops). Negative circuits can bring about oscillatory dynamics and positive ones to multi-stable states behavior. These circuits command the dynamics of molecular systems within the cells. To check their consequence on the dynamics, the Boolean technique permits in silico perturbations of nodes, i.e., forcing them to stay very fixed coequal to loss (LoF) or gain of function (GoF) experiments and having by interaction perturbations. Node or interaction perturbations can undermine the consequence of an operational circuit evidencing their precise role within the dynamics.

### 2.3. Database and Tools

We constructed our Boolean model utilizing GINsim 3.0.0b, a Java-based software and freely available for the researchers (http://ginsim.org/downloads) [29]. The model was further developed based on the literature and using various public databases such as BioGRID 3.5 (https://thebiogrid.org/) [30], TargetScanHuman 7.1 (http://www.targetscan.org/vert_71/) [31] and miRTargetLink (https://ccb-web.cs.uni-saarland.de/mirtargetlink/index.php) [32].

The GINsim model file is available in the Data File S1.

## 3. Results

**Figure 2:**
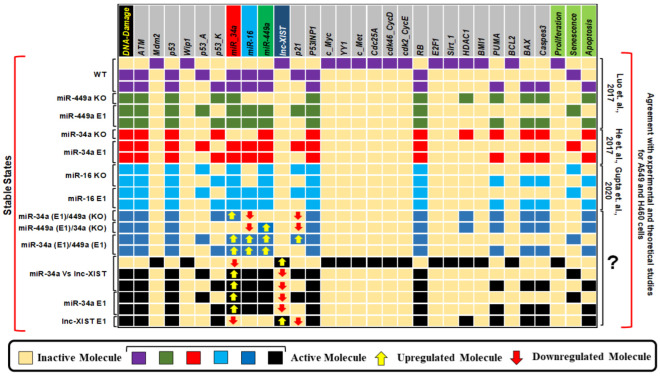
**Model results.** WT case states and results of Gain-of-Function (GoF) and Loss-of-Function (LoF) perturbations coordinate to the referential studies operated in this work. The question mark denotes model predictions. Ectopic expression (E1) describes gain of function and Knockout (KO) loss of function of the similar model component. Yellow cells denote inactivation (OFF), whereas colored cells such as: purple, green, red, light blue, dark blue and black denote activation (ON). The stable states were pinpointed for different networking scenarios: WT, miR-449a KO, miR-449a E1, miR-34a KO, miR-34a E1, miR-16 KO, miR-16 E1, miR-34a E1 along with miR-449a KO, miR-449a E1 along with miR-34a KO. Cells in dark blue color and black color that describe the model predictions and indicated by the question mark such as mention previously, in this way, miR-34a Vs lncRNA XIST, Negative correlation between miR-34a and lncRNA XIST in DNA damage response, miR-34a E1, and lncRNA XIST E1. Red arrows designate down-regulation, whereas yellow arrows convey up-regulation. The left-most queue exhibits the input DNA Damage and the right-most queues demonstrate the model developments: proliferation, apoptosis, and senescence. Per line symbolizes a unique fixed point be compatible with the input.

The fixed points (also known as stable states) for the wild-type case (WT) and mutants are illustrated in Figure 2 together with experimental results and references. The input of the model is DNA damage which can be ON or OFF. Each line of the figure is a unique fixed point of the network. The first 3 lines correspond to the WT. The first fixed point of the WT is a proliferative state rising from the lack of DNA damage and is defined by activation of cell cycle regulators and inactivation of cell cycle inhibitors: E2F1, Cdk46-CycD, Cdk2-CycE c_Myc, Cdc25A, and lncRNA XIST. The next two fixed points of the WT for when DNA damage ON correlated to the bistability involving senescence and apoptosis. Bistability is a stochastic behavior of the model dynamics where two different fixed points are randomly selected from the same initial condition. The probabilities of these fixed points are not necessarily equal. This bistability is regulated by the p53-A/p53-K circuit [33], which was proposed in previous works [13,17,33,34,35]. The remaining phenotypes in Figure 2 follow from mutations that will be discussed below.

In Table 1 we pointed the experiments used as influential references to our study (for more details see Figure S1. The model was fine-tuned to come up with the knowledge provided by each study (see section Methods for more details about the biochemical information and also the network construction). Some studies focused only on the interaction between the lncRNA XIST and one miRNA, while other studies considered more interacting miRNAs with lncRNA XIST (Table 1 and Figure 2). Here we resort to many of those studies.

First, we interrogated how perturbations of the network connecting the non-coding RNAs affect cell fate (results are presented in Figure 2). To do that, we considered the experiments in Table 1 that focused on the lncRNA XIST and just one or more of the other miRNAs. For example, the investigation of the particular regulatory role of a single miRNA was conducted making an *in-silico* knockout (KO) of the others. We confirmed that different perturbations of miR-449a and miR-34a induce different phenotypes as observed in experiments using A549 and H460 cells by Luo et al. [38] and He et al. [36]. Other perturbations correspond to predictions, as they were not observed experimentally. For example, the combined perturbation of miR-449a overexpression (E1) with miR-34a KO, or the inverse perturbation, induced only apoptosis. However, the overexpression of both nodes generates a bistable state of senescence and apoptosis. This result is experimentally supported by the fact that both miR-449a and miR-34a directly inhibit the expressions of c-Myc, HDAC1, and Bcl2, which initiates the activation of p16 and p21, leading to cycle arrest. See Figure 2.

GINsim allows us to recognize functional circuits within the network, i.e., circuits that actively sustain the dynamics of network (see Section 2). We found 27 positive and 14 negative circuits (Table 2). More details about the functional circuits of the Boolean model can be found in Table S2. However, we elected only 25 of those circuits containing an utmost of four components, a number of them were already studied experimentally. Only twelve, out of these 25, involving miR-34a, miR-16 and miR-449a and/or lncRNA XIST are unknown. The biochemical interactions explaining these 12 positive circuits are well-known within the literature for NSCLC cells (Table 3), however their performance in the G1/S checkpoint is yet to be determined. Then, as observed experimentally NSCLC, we investigated through in-silico perturbations whether these circuits were important to manage the WT-case. We executed edge perturbations on the circuits (see Table 4). This sort of perturbation is challenging to be obtained experimentally, nonetheless, they are truly valuable to specify circuit significance. First, we select an edge and eliminate it. For example, for the circuit miR-34a/YY1/lncRNA XIST, we removed the interaction between miR-34a and lncRNA XIST. We obtained in this case as output states, senescence and apoptosis and then, removing the interaction between YY1 and lncRNA XIST, we got two apoptotic and one senescence states. And at last, removing the interaction between lncRNA XIST and miR-34a, we obtained senescence and apoptosis. Using this technique, we perturbed all 12 circuits. As can be seen in Table 4, we found that the interaction between YY1 and lncRNA XIST (belonging to the circuits miR-34a/YY1/lncRNA XIST, YY1/lncRNA XIST/miR-34a/c-Myc and miR-449a/c-Myc/YY1/lncRNA XIST) is responsible for generating apoptotic and one senescent state. Whereas, the interaction between Rb and c-Myc in circuits CDK4-6-CycD/RB/c-Myc/miR-16 and CDK2-CycE/RB/c-Myc/miR-16 addresses for two apoptotic, one senescent and one undefined state. In this way, we determined that only five, out of 12 circuits, could produce multistability (Table 4, highlighted in bold fonts).

## 4. Discussion

It is well recognized that RNAs interaction networks (lncRNA-miRNA-mRNA) play an essential role in regulatory mechanisms and cancer [1,2]. In this context, lncRNA XIST has been studied in several cancers including NSCLC. It has been observed that lncRNA applies its biological influences on cancer by sponging or indirect interactions with XIST miRNAs. MiR-34a, miR-449a, and miR-16 regulate cell cycle progression by targeting CDK4,6-CyclinD and CDK2-Cyclin E complexes and the lncRNA XIST sponges these miRNAs. In the current study, based on a previously published work [17], we presented a Boolean model of the plausible network connecting lncRNA XIST/miR-34a/miR-449a and miR-16 in NSCLC (see Figure 1).

Lately, Zhang et al. [14] and Zhou et al. [15] demonstrated that lncRNA XIST can regulate proliferation and tumor formation by targeting miR-449a/miR-16 in NSCLC. On the other hand, Liu et al. [16], pointed the role of lncRNA XIST/miR-34a axis in thyroid cancer. They further demonstrated that lncRNA XIST was significantly up-regulated and miR-34a was under expressed in Thyroid cancer [16]. Moreover, lncRNA XIST knockdown inhibits cell proliferation in-vivo and in-vitro [16] in thyroid cancer cell lines. Interestingly, miR-34a is downregulated in NSCLC [36]. However, no other investigation in the literature analyzed the role of the miR-34a/lncRNA XIST axis on NSCLC. Thus, this finding detected in Liu et al. [16] study permits us to predict the mechanisms identified for miR-34a/lncRNA XIST in thyroid cancer cells that might also be observed in NSCLC cells.

**Figure 3:**
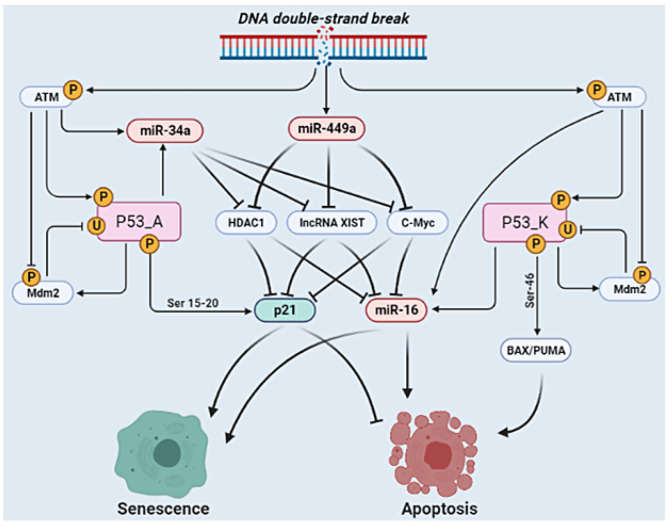
**Molecular mechanisms of p21 and miR-16 through the miR-449a in response to DNA damage in NSCLC.** DNA double-strand break (DSB) can be caused by the radiomimetic chemicals or reactive oxygen species (ROS) or radiation, which triggers autophosphorylation of ATM at serine 1981 by initiating its kinase activity [64]. Downstream phosphorylation on the ATM pathway drives the activation of p53 in response to DNA damage. ATM directly induces Mdm2 phosphorylation, thereby reducing the activity of Mdm2 and resulting in strong induction of p53. Activated p53 induces Mdm2 which inhibits p53 by ubiquitination [49]. Within the model, based on the different phosphorylation events that p53 controls, it is described by two variables: p53-A and p53-K. p53-A represents p53 phosphorylated at serine 15 and serine 20 that induce p21 expression for the cell cycle arrest or senescence, whereas p53-K specifies further phosphorylation at serine 46 that modulates Bax and/or PUMA expression for the apoptosis (see [33] for more information on p53-A/p53-K function). Activated ATM/p53 can induce miR-34a [50,65] and miR-16 [66,67] expression in DNA damage response. On the other hand, miR-449a induced through the DNA damage as suggested by Mao et al. [21]. HDAC1, lncRNA XIST and C-Myc are well-known inhibitors of p21 [68,69,70] and miR-16 [15,71,72]. Interestingly, HDAC1, lncRNA XIST, and C-Myc are directly targeted by the miR-449a [14,21,73] and miR-34a [16,36,74]. Consequently, miR-449a can regulate p21 expression and miR-16 activity through the suppression of HDAC1, lncRNA XIST, and C-Myc. Once p21 expression and/or miR-16 is activated, it can trigger senescence and/or apoptosis in NSCLC at the G1/S checkpoint. Arrowhead edge represents activation. Whereas, a hammer-head edge represents suppression, respectively. Detailed molecular mechanism of DNA damage of the model see our previous publish study [17] and also the latest reviewed article by Huang et al. [75].

Thus, to advance our understanding of the synergistic coordination of cell fate determination in NSCLC cells within a non-coding RNA interaction framework, a mechanistic understanding of this development is needed. Therefore, a Boolean model was constructed that observed in the experimental outcomes regarding the action of these molecules at the G1/S checkpoint in NSCLC cells. In the loss of DNA damage, the model defines only a unique fixed point represented by a proliferative phenotype that is expected. In contrast, in the occurrence of DNA damage, two fixed points (p53-dependent cell fates such as senescence or apoptosis) were obtained. Certainly, in NSCLC both phenotypes are recognized in response to DNA damage. In addition, the Boolean model was investigated through GoF and LoF perturbations of its variables corresponding to the experimental outcomes (see Table 1) from multiple experimental studies [14,15,36,37,38,39,40] which examined the interaction between lncRNA XIST and miRNAs (miR-449a, miR-16, and miR-34a) in NSCLC cells. Previously, we have shown that miR-34a can regulate miR-16 expression by targeting HDAC1 and c-Myc [17]. Remarkable, we detected that miR-449a can regulate miR-16 as well as p21 expression through HDAC1, c-Myc, and lncRNA XIST. Additionally, we found that miR-34a can control miR-16 and p21 expression via lncRNA XIST knockdown (for more details, see Figure 2 and Figure 3). In addition, in gene regulatory networks (GRN), biological circuits play a vital role. Certainly, GRN is a mixture of various circuits that can interpret the dynamics of a biological system [62,63]. In this regard, we found twelve new positive circuits (see Table 2), which by perturbation analysis suggested that only five of these circuits affect (see Table 4, highlighted in bold fonts) cell fate decisions. We also provided evidence of these circuits’ presence according to NSCLC (for more details see Table 3).

## 5. Conclusions

In summary, we proposed an interaction network connecting several non-coding RNAs and predicted that miR-449a can control miR-16 and p21 expression through HDAC1, c-Myc, and lncRNA XIST. Our approach may contribute to suggest alternative therapeutic strategies for cancer by targeting non-coding RNAs embedded in checkpoint regulatory networks.

## Figures and Tables

**Table 1:** Referential experimental studies used to develop the network. E1 represents gain of function (GoF) and KO stands for LoF of the corresponding molecules.

Stimulus/Perturbations	Model Response/Phenotype	Cell Lines	References
miR-34a in the absence of DNA Damage	Proliferation	A549, H460	[36]
miR-34a in the presence of DNA Damage	Triggers senescence and apoptosis	A549, H460	[36]
miR-34a Vs c-Myc in response to DNA Damage	Negative correlation	A549, H460	[36]
miR-34a E1	Induces Senescence and Apoptosis	A549, H460	[36]
c-Myc KO	Activating Senescence and Apoptosis	A549, H460	[36]
miR-16 E1 and RB E1	Induces Senescence and Apoptosis	A549	[37]
miR-16 E1 and RB KO	Induces only Apoptosis	A549	[37]
miR-16 E1	Activating Senescence and Apoptosis	A549	[37]
miR-449a Vs C-Met in response to DNA Damage	Negative correlation	A549	[38]
miR-449a KO	Proliferation	A549	[38]
miR-449a E1	Induces Senescence and Apoptosis	A549	[38]
C-Met KO	Activating Senescence and Apoptosis	A549	[38]
miR-34a E1 and miR-16 E1	Activating Senescence and Apoptosis	A549	[39]
miR-34a E1 and RB E1	Induces Senescence and Apoptosis	A549	[39]
miR-34a KO and miR-16 KO	Induces only Apoptosis	A549	[39]
RB E1	Induces Senescence and Apoptosis	A549	[39]
miR-34a KO and miR-449a KO	Proliferation	A549	[40]
miR-34a E1 and miR-449a E1	Induces Senescence and Apoptosis	A549	[40]
miR-449a Vs lncRNA XIST in DNA Damage Response	Negative correlation	A549, H460	[14]
miR-449a E1	Activating Senescence and Apoptosis	A549, H460	[14]
lncRNA XIST KO	Induces Senescence and Apoptosis	A549, H460	[14]
miR-16 Vs lncRNA XIST in DNA Damage Response	Negative correlation	A549	[15]
miR-16 E1	Triggers senescence and apoptosis	A549	[15]
lncRNA XIST KO	Induces Senescence and Apoptosis	A549	[15]

**Table 2:** Functional circuits in the network and experimental observations. Circuits for which no experimental data were found are indicated by question marks (?) and highlighted in bold fonts.

Number	Circuits	Reference
	**Positive**	
1	c-Myc/E2F1	[41]
2	p21/Caspase3	[42]
3	ATM/miR-34a/HDAC1	[34]
4	**miR-449a/lncRNA XIST**	?
5	**miR-16/lncRNA XIST**	?
6	**miR-34a/lncRNA XIST**	?
7	**p53/miR-34a/C-Met**	?
8	**miR-34a/YY1/lncRNA XIST**	?
9	**YY1/p53/miR-34a/c-Myc**	?
10	**YY1/lncRNA XIST/miR-34a/c-Myc**	?
11	**CDK4-6-CycD/RB/c-Myc/miR-16**	?
12	**CDK2-CycE/RB/c-Myc/miR-16**	?
13	**p21/c-Myc/YY1/p53**	?
14	**p21/c-Myc/c-Met/p53**	?
15	**miR-449a/c-Myc/YY1/lncRNA XIST**	?
16	p53-K/p53-A	[33]
17	p53/miR-34a/YY1	[43]
18	p53/miR-34a/Sirt1	[44]
19	E2F1/ATM	[45]
20	E2F1/CDK2-CycE/RB	[46]
21	p21/c-Myc	[47]
	**Negative**	
22	p53/Mdm2	[48]
23	p53INP1/p53-A	[33]
24	ATM/miR-34a/E2F1	[34]
25	E2F1/Sirt1	[49]

**Table 3:** The evidence of the biochemical interactions defining the positive circuits is well-characterized in the literature.

Positive Circuits	Circuit Molecules	Target	Direct/indirect Interaction	References
miR-449a/lncRNA XIST	miR-449a	lncRNA XIST	Direct inhibition	[14]
	lncRNA XIST	miR-449a	Direct inhibition	[14]
miR-16/lncRNA XIST	miR-16	lncRNA XIST	Direct inhibition	[15]
	lncRNA XIST	miR-16	Direct inhibition	[15]
miR-34a/lncRNA XIST	miR-34a	lncRNA XIST	Direct inhibition	[16]
	lncRNA XIST	miR-34a	Direct inhibition	[16]
p53/miR-34a/C-Met	p53	miR-34a	Direct activation	[50]
	miR-34a	C-Met	Direct inhibition	[51]
	C-Met	p53	Direct inhibition	[52]
miR-34a/YY1/lncRNA XIST	miR-34a	YY1	Direct inhibition	[43]
	YY1	lncRNA XIST	Direct activation	[22]
	lncRNA XIST	miR-34a	Direct inhibition	[16]
YY1/p53/miR-34a/c-Myc	YY1	p53	Direct inhibition	[25]
	p53	miR-34a	Direct activation	[50]
	miR-34a	c-Myc	Direct inhibition	[36]
	c-Myc	YY1	Direct activation	[53]
YY1/lncRNA XIST/miR-34a/c-Myc	YY1	lncRNA XIST	Direct activation	[22]
	lncRNA XIST	miR-34a	Direct inhibition	[16]
	miR-34a	c-Myc	Direct inhibition	[36]
	c-Myc	YY1	Direct activation	[53]
CDK4-6-CycD/RB/c-Myc/miR-16	CDK4-6-CycD	RB	Direct inhibition	[54]
	RB	c-Myc	Direct inhibition	[55]
	c-Myc	miR-16	Direct inhibition	[56]
	miR-16	CDK4-6-CycD	Direct inhibition	[57]
CDK2-CycE/RB/c-Myc/miR-16	CDK2-cycE	RB	Direct inhibition	[54]
	RB	c-Myc	Direct inhibition	[55]
	c-Myc	miR-16	Direct inhibition	[56]
	miR-16	CDK2-CycE	Direct inhibition	[57]
p21/c-Myc/YY1/p53	p21	c-Myc	Direct inhibition	[58]
	c-Myc	YY1	Direct activation	[53]
	YY1	p53	Direct inhibition	[25]
	p53	p21	Direct activation	[59]
p21/c-Myc/c-Met/p53	p21	c-Myc	Direct inhibition	[58]
	c-Myc	C-Met	Direct activation	[60]
	C-Met	p53	Direct inhibition	[61]
	p53	p21	Direct activation	[59]
miR-449a/c-Myc/YY1/lncRNA XIST	miR-449a	c-Myc	Direct inhibition	[21]
	c-Myc	YY1	Direct activation	[53]
	YY1	lncRNA XIST	Direct activation	[22]
	lncRNA XIST	miR-449a	Direct inhibition	[14]

**Table 4:** Circuits and their corresponding perturbations that were found to responsible for three states or more are highlighted in bold fonts. Point to be noted here other 1 state means undefined state (for more detail see the text).

Positive Circuits	Removed Interactions	Phenotype Observation
miR-449a/lncRNA XIST	miR-449a/lncRNA XIST	Senescence and Apoptosis
	lncRNA XIST/miR-449a	Senescence and Apoptosis
miR-16/lncRNA XIST	miR-16/lncRNA XIST	Senescence and Apoptosis
	lncRNA XIST/miR-16	Senescence and Apoptosis
miR-34a/lncRNA XIST	miR-34a/lncRNA XIST	Senescence and Apoptosis
	lncRNA XIST/miR-34a	Senescence and Apoptosis
p53/miR-34a/C-Met	p53/miR-34a	Senescence and Apoptosis
	miR-34a/C-Met	Senescence and Apoptosis
	C-Met/p53	Senescence and Apoptosis
**miR-34a/YY1/lncRNA XIST**	miR-34a/YY1	Senescence and Apoptosis
	**YY1/lncRNA XIST**	Two states for Apoptosis and one state for senescence
	lncRNA XIST/miR-34a	Senescence and Apoptosis
YY1/p53/miR-34a/c-Myc	YY1/p53	Senescence and Apoptosis
	p53/miR-34a	Senescence and Apoptosis
	miR-34a/c-Myc	Senescence and Apoptosis
	c-Myc/YY1	Senescence and Apoptosis
**YY1/lncRNA XIST/miR-34a/c-Myc**	**YY1/lncRNA XIST**	Two states for Apoptosis and one state for senescence
	lncRNA XIST/miR-34a	Senescence and Apoptosis
	miR-34a/c-Myc	Senescence and Apoptosis
	c-Myc/YY1	Senescence and Apoptosis
**CDK4-6-CycD/RB/c-Myc/miR-16**	CDK4-6-CycD/RB	Senescence and Apoptosis
	**RB/c-Myc**	4 States (2 apoptotic and 1 senescence and other 1)
	c-Myc/miR-16	Senescence and Apoptosis
	miR-16/CDK4-6-CycD	Senescence and Apoptosis
**CDK2-CycE/RB/c-Myc/miR-16**	CDK2-CycE/RB	Senescence and Apoptosis
	**RB/c-Myc**	4 States (2 apoptotic and 1 senescence and other 1)
	c-Myc/miR-16	Senescence and Apoptosis
	miR-16/CDK2-CycE	Senescence and Apoptosis
p21/c-Myc/YY1/p53	p21/c-Myc	Senescence and Apoptosis
	c-Myc/YY1	Senescence and Apoptosis
	YY1/p53	Senescence and Apoptosis
	p53/p21	Senescence and Apoptosis
p21/c-Myc/c-Met/p53	p21/c-Myc	Senescence and Apoptosis
	c-Myc/C-Met	Senescence and Apoptosis
	C-Met/p53	Senescence and Apoptosis
	p53/p21	Senescence and Apoptosis
**miR-449a/c-Myc/YY1/lncRNA XIST**	miR-449a/c-Myc	Senescence and Apoptosis
	c-Myc/YY1	Senescence and Apoptosis
	**YY1/lncRNA XIST**	Two states for Apoptosis and one state for senescence
	lncRNA XIST/miR-449a	Senescence and Apoptosis

## Data Availability

Data is contained within the article or supplementary material.

## Supplementary Materials

The following are available online at https://www.mdpi.com/article/10.3390/biology11040480/s1, Figure S1: The experiments used as major references to our study, Data File S1: The GINsim 3.0.0b code of the model. Table S1: Official names of the model elements and logical rules. Table S2: Functional circuits of the Boolean model.

## References

[1] Wang K.C., Chang H.Y. (2011). Molecular mechanisms of long noncoding RNAs. Mol. Cell.

[2] Prensner J.R., Chinnaiyan A.M. (2011). The emergence of lncRNAs in cancer biology. Cancer Discov..

[3] do Valle Í.F., Menichetti G., Simonetti G., Bruno S., Zironi I., Durso D.F., Mombach J.C.M., Martinelli G., Castellani G., Remondini D. (2018). Network integration of multi-tumour omics data suggests novel targeting strategies. Nat. Commun..

[4] Chen D., Ju H., Lu Y., Chen L., Zeng Z., Zhang D., Luo H., Wang F., Qiu M., Wang D. (2016). Long non-coding RNA XIST regulates gastric cancer progression by acting as a molecular sponge of miR-101 to modulate EZH2 expression. J. Exp. Clin. Cancer Res..

[5] Cheng Z., Li Z., Ma K., Li X., Tian N., Duan J., Xiao X., Wang Y. (2017). Long non-coding RNA XIST promotes glioma tumorigenicity and angiogenesis by acting as a molecular sponge of miR-429. J. Cancer.

[6] Xu X., Zhou X., Chen Z., Gao C., Zhao L., Cui Y. (2020). Silencing of lncRNA XIST inhibits non-small cell lung cancer growth and promotes chemosensitivity to cisplatin. Aging (Albany NY).

[7] Tian L.J., Wu Y.P., Wang D., Zhou Z.H., Xue S.B., Zhang D.Y., Wei Y.G., Liu W. (2019). Upregulation of long noncoding RNA (lncRNA) X-inactive specific transcript (XIST) is associated with cisplatin resistance in non-small cell lung cancer (NSCLC) by downregulating microRNA-144-3p. Med. Sci. Monit. Int. Med. J. Exp. Clin. Res..

[8] Marshall E.A., Stewart G.L., Sage A.P., Lam W.L., Brown C.J. (2019). Beyond sequence homology: Cellular biology limits the potential of XIST to act as a miRNA sponge. PLoS ONE.

[9] Li C., Wan L., Liu Z., Xu G., Wang S., Su Z., Zhang Y., Zhang C., Liu X., Lei Z. (2018). Long non-coding RNA XIST promotes TGF-*β*-induced epithelial-mesenchymal transition by regulating miR-367/141-ZEB2 axis in non-small-cell lung cancer. Cancer Lett..

[10] Wang X., Zhang G., Cheng Z., Dai L., Jia L., Jing X., Wang H., Zhang R., Liu M., Jiang T. (2018). Knockdown of LncRNA-XIST suppresses proliferation and TGF-*β*1-induced EMT in NSCLC through the Notch-1 pathway by regulation of miR-137. Genet. Test. Mol. Biomark..

[11] Wang J., Cai H., Dai Z., Wang G. (2019). Down-regulation of lncRNA XIST inhibits cell proliferation via regulating miR-744/RING1 axis in non-small cell lung cancer. Clin. Sci..

[12] Gupta S., Silveira D.A., Mombach J.C.M. (2018). Modeling the role of microRNA-449a in the regulation of the G2/M cell cycle checkpoint in prostate LNCaP cells under ionizing radiation. PLoS ONE.

[13] Gupta S., Silveira D.A., Mombach J.C.M. (2020). Towards DNA-damage induced autophagy: A Boolean model of p53-induced cell fate mechanisms. DNA Repair.

[14] Zhang Y., Li X., Hou Y., Fang N., You J., Zhou Q. (2017). The lncRNA XIST exhibits oncogenic properties via regulation of miR-449a and Bcl-2 in human non-small cell lung cancer. Acta Pharmacol. Sin..

[15] Zhou X., Xu X., Gao C., Cui Y. (2019). XIST promote the proliferation and migration of non-small cell lung cancer cells via sponging miR-16 and regulating CDK8 expression. Am. J. Transl. Res..

[16] Liu H., Deng H., Zhao Y., Li C., Liang Y. (2018). LncRNA XIST/miR-34a axis modulates the cell proliferation and tumor growth of thyroid cancer through MET-PI3K-AKT signaling. J. Exp. Clin. Cancer Res..

[17] Gupta S., Silveira D.A., Barbé-Tuana F.M., Mombach J.C.M. (2020). Integrative data modeling from lung and lymphatic cancer predicts functional roles for miR-34a and miR-16 in cell fate regulation. Sci. Rep..

[18] Lizé M., Klimke A., Dobbelstein M. (2011). MicroRNA-449 in cell fate determination. Cell Cycle.

[19] Lv J., Zhang Z., Pan L., Zhang Y. (2019). MicroRNA-34/449 family and viral infections. Virus Res..

[20] Aqeilan R., Calin G., Croce C. (2010). miR-15a and miR-16-1 in cancer: Discovery, function and future perspectives. Cell Death Differ..

[21] Mao A., Zhao Q., Zhou X., Sun C., Si J., Zhou R., Gan L., Zhang H. (2016). MicroRNA-449a enhances radiosensitivity by downregulation of c-Myc in prostate cancer cells. Sci. Rep..

[22] Makhlouf M., Ouimette J.F., Oldfield A., Navarro P., Neuillet D., Rougeulle C. (2014). A prominent and conserved role for YY1 in Xist transcriptional activation. Nat. Commun..

[23] Nie J., Ge X., Geng Y., Cao H., Zhu W., Jiao Y., Wu J., Zhou J., Cao J. (2015). miR-34a inhibits the migration and invasion of esophageal squamous cell carcinoma by targeting Yin Yang-1. Oncol. Rep..

[24] Cai Y., Jiang C., Zhu J., Xu K., Ren X., Xu L., Hu P., Wang B., Yuan Q., Guo Y. (2019). miR-449a inhibits cell proliferation, migration, and inflammation by regulating high-mobility group box protein 1 and forms a mutual inhibition loop with Yin Yang 1 in rheumatoid arthritis fibroblast-like synoviocytes. Arthritis Res. Ther..

[25] Grönroos E., Terentiev A.A., Punga T., Ericsson J. (2004). YY1 inhibits the activation of the p53 tumor suppressor in response to genotoxic stress. Proc. Natl. Acad. Sci. USA.

[26] Jung H.Y., Joo H.J., Park J.K., Kim Y.H. (2012). The blocking of c-Met signaling induces apoptosis through the increase of p53 protein in lung cancer. Cancer Res. Treat. Off. J. Korean Cancer Assoc..

[27] Imani S., Wu R.C., Fu J. (2018). MicroRNA-34 family in breast cancer: From research to therapeutic potential. J. Cancer.

[28] Abou-Jaoudé W., Traynard P., Monteiro P.T., Saez-Rodriguez J., Helikar T., Thieffry D., Chaouiya C. (2016). Logical modeling and dynamical analysis of cellular networks. Front. Genet..

[29] Naldi A., Hernandez C., Abou-Jaoudé W., Monteiro P.T., Chaouiya C., Thieffry D. (2018). Logical modeling and analysis of cellular regulatory networks with ginsim 3.0. Front. Physiol..

[30] Chatr-Aryamontri A., Oughtred R., Boucher L., Rust J., Chang C., Kolas N.K., O’Donnell L., Oster S., Theesfeld C., Sellam A. (2017). The BioGRID interaction database: 2017 update. Nucleic Acids Res..

[31] Agarwal V., Bell G.W., Nam J.W., Bartel D.P. (2015). Predicting effective microRNA target sites in mammalian mRNAs. elife.

[32] Hamberg M., Backes C., Fehlmann T., Hart M., Meder B., Meese E., Keller A. (2016). MiRTargetLink—miRNAs, genes and interaction networks. Int. J. Mol. Sci..

[33] Zhang X.P., Liu F., Wang W. (2011). Two-phase dynamics of p53 in the DNA damage response. Proc. Natl. Acad. Sci. USA.

[34] Gupta S., Silveira D.A., Mombach J.C.M. (2020). ATM/miR-34a-5p axis regulates a p21-dependent senescence-apoptosis switch in non-small cell lung cancer: A Boolean model of G1/S checkpoint regulation. FEBS Lett..

[35] Silveira D.A., Gupta S., Mombach J.C.M. (2020). p53/E2F1/miR-25 axis regulates apoptosis induction in glioblastoma cells: A qualitative model. J. Phys. Complex..

[36] He X., Yang A., McDonald D.G., Riemer E.C., Vanek K.N., Schulte B.A., Wang G.Y. (2017). MiR-34a modulates ionizing radiation-induced senescence in lung cancer cells. Oncotarget.

[37] Bandi N., Zbinden S., Gugger M., Arnold M., Kocher V., Hasan L., Kappeler A., Brunner T., Vassella E. (2009). miR-15a and miR-16 are implicated in cell cycle regulation in a Rb-dependent manner and are frequently deleted or down-regulated in non–small cell lung cancer. Cancer Res..

[38] Luo W., Huang B., Li Z., Li H., Sun L., Zhang Q., Qiu X., Wang E. (2013). MicroRNA-449a is downregulated in non-small cell lung cancer and inhibits migration and invasion by targeting c-Met. PLoS ONE.

[39] Bandi N., Vassella E. (2011). miR-34a and miR-15a/16 are co-regulated in non-small cell lung cancer and control cell cycle progression in a synergistic and Rb-dependent manner. Mol. Cancer.

[40] Chevalier B., Adamiok A., Mercey O., Revinski D.R., Zaragosi L.E., Pasini A., Kodjabachian L., Barbry P., Marcet B. (2015). miR-34/449 control apical actin network formation during multiciliogenesis through small GTPase pathways. Nat. Commun..

[41] Coller H.A., Forman J.J., Legesse-Miller A. (2007). “Myc’ed messages”: Myc induces transcription of E2F1 while inhibiting its translation via a microRNA polycistron. PLoS Genet..

[42] Zhang Y., Fujita N., Tsuruo T. (1999). Caspase-mediated cleavage of p21 Waf1/Cip1 converts cancer cells from growth arrest to undergoing apoptosis. Oncogene.

[43] Chen Q.R., Yu L.R., Tsang P., Wei J.S., Song Y.K., Cheuk A., Chung J.Y., Hewitt S.M., Veenstra T.D., Khan J. (2011). Systematic proteome analysis identifies transcription factor YY1 as a direct target of miR-34a. J. Proteome Res..

[44] Yamakuchi M., Ferlito M., Lowenstein C.J. (2008). miR-34a repression of SIRT1 regulates apoptosis. Proc. Natl. Acad. Sci. USA.

[45] Lin W.C., Lin F.T., Nevins J.R. (2001). Selective induction of E2F1 in response to DNA damage, mediated by ATM-dependent phosphorylation. Genes Dev..

[46] Pomerening J.R. (2009). Positive-feedback loops in cell cycle progression. FEBS Lett..

[47] Jänicke R.U., Sohn D., Essmann F., Schulze-Osthoff K. (2007). The multiple battles fought by anti-apoptotic p21. Cell Cycle.

[48] Bar-Or R.L., Maya R., Segel L.A., Alon U., Levine A.J., Oren M. (2000). Generation of oscillations by the p53-Mdm2 feedback loop: A theoretical and experimental study. Proc. Natl. Acad. Sci. USA.

[49] Wang C., Chen L., Hou X., Li Z., Kabra N., Ma Y., Nemoto S., Finkel T., Gu W., Cress W.D. (2006). Interactions between E2F1 and SirT1 regulate apoptotic response to DNA damage. Nat. Cell Biol..

[50] Raver-Shapira N., Marciano E., Meiri E., Spector Y., Rosenfeld N., Moskovits N., Bentwich Z., Oren M. (2007). Transcriptional activation of miR-34a contributes to p53-mediated apoptosis. Mol. Cell.

[51] Cortez M.A., Valdecanas D., Niknam S., Peltier H.J., Diao L., Giri U., Komaki R., Calin G.A., Gomez D.R., Chang J.Y. (2015). In vivo delivery of miR-34a sensitizes lung tumors to radiation through RAD51 regulation. Mol. Ther.-Nucleic Acids.

[52] Liu Y., Liu J.H., Chai K., Tashiro S.I., Onodera S., Ikejima T. (2013). Inhibition of c-M et promoted apoptosis, autophagy and loss of the mitochondrial transmembrane potential in oridonin-induced A 549 lung cancer cells. J. Pharm. Pharmacol..

[53] Shrivastava A., Yu J., Artandi S., Calame K. (1996). YY1 and c-Myc associate in vivo in a manner that depends on c-Myc levels. Proc. Natl. Acad. Sci. USA.

[54] Giacinti C., Giordano A. (2006). RB and cell cycle progression. Oncogene.

[55] Zhao X., Day M.L. (2001). RB activation and repression of C-MYC transcription precede apoptosis of human prostate epithelial cells. Urology.

[56] Williams M., Cheng Y.Y., Kirschner M.B., Sarun K.H., Schelch K., Winata P., McCaughan B., Kao S., Van Zandwijk N., Reid G. (2019). Transcriptional suppression of the miR-15/16 family by c-Myc in malignant pleural mesothelioma. Oncotarget.

[57] Liu Q., Fu H., Sun F., Zhang H., Tie Y., Zhu J., Xing R., Sun Z., Zheng X. (2008). miR-16 family induces cell cycle arrest by regulating multiple cell cycle genes. Nucleic Acids Res..

[58] Vigneron A., Cherier J., Barré B., Gamelin E., Coqueret O. (2006). The cell cycle inhibitor p21waf1 binds to the myc and cdc25A promoters upon DNA damage and induces transcriptional repression. J. Biol. Chem..

[59] Shamloo B., Usluer S. (2019). p21 in cancer research. Cancers.

[60] Gabay M., Li Y., Felsher D.W. (2014). MYC activation is a hallmark of cancer initiation and maintenance. Cold Spring Harb. Perspect. Med..

[61] Mikami K., Medova M., Nisa L., Francica P., Glück A.A., Tschan M., Blaukat A., Bladt F., Aebersold D., Zimmer Y. (2015). Impact of p53 Status on Radiosensitization of Tumor Cells by MET Inhibition—Associated Checkpoint Abrogation. Mol. Cancer Res..

[62] Gupta S., Hashimoto R.F. (2022). Dynamical Analysis of a Boolean Network Model of the Oncogene Role of lncRNA ANRIL and lncRNA UFC1 in Non-Small Cell Lung Cancer. Biomolecules.

[63] Silveira D.A., Gupta S., Mombach J.C.M. (2020). Systems biology approach suggests new miRNAs as phenotypic stability factors in the epithelial–mesenchymal transition. J. R. Soc. Interface.

[64] Bakkenist C.J., Kastan M.B. (2003). DNA damage activates ATM through intermolecular autophosphorylation and dimer dissociation. Nature.

[65] Salzman D.W., Nakamura K., Nallur S., Dookwah M.T., Metheetrairut C., Slack F.J., Weidhaas J.B. (2016). miR-34 activity is modulated through 5’-end phosphorylation in response to DNA damage. Nat. Commun..

[66] Zhang X., Wan G., Berger F.G., He X., Lu X. (2011). The ATM kinase induces microRNA biogenesis in the DNA damage response. Mol. Cell.

[67] Suzuki H.I., Yamagata K., Sugimoto K., Iwamoto T., Kato S., Miyazono K. (2009). Modulation of microRNA processing by p53. Nature.

[68] Wang L., Li H., Ren Y., Zou S., Fang W., Jiang X., Jia L., Li M., Liu X., Yuan X. (2016). Targeting HDAC with a novel inhibitor effectively reverses paclitaxel resistance in non-small cell lung cancer via multiple mechanisms. Cell Death Dis..

[69] Xu T., Jiang W., Fan L., Gao Q., Li G. (2017). Upregulation of long noncoding RNA Xist promotes proliferation of osteosarcoma by epigenetic silencing of P21. Oncotarget.

[70] Jeong J.H., Kang S.S., Park K.K., Chang H.W., Magae J., Chang Y.C. (2010). p53-independent induction of G1 arrest and p21WAF1/CIP1 expression by ascofuranone, an isoprenoid antibiotic, through downregulation of c-Myc. Mol. Cancer Ther..

[71] Sampath D., Liu C., Vasan K., Sulda M., Puduvalli V.K., Wierda W.G., Keating M.J. (2012). Histone deacetylases mediate the silencing of miR-15a, miR-16, and miR-29b in chronic lymphocytic leukemia. Blood J. Am. Soc. Hematol..

[72] Zhang X., Chen X., Lin J., Lwin T., Wright G., Moscinski L., Dalton W., Seto E., Wright K., Sotomayor E. (2012). Myc represses miR-15a/miR-16-1 expression through recruitment of HDAC3 in mantle cell and other non-Hodgkin B-cell lymphomas. Oncogene.

[73] Noonan E., Place R., Pookot D., Basak S., Whitson J.M., Hirata H., Giardina C., Dahiya R. (2009). miR-449a targets HDAC-1 and induces growth arrest in prostate cancer. Oncogene.

[74] Zhao J., Lammers P., Torrance C.J., Bader A.G. (2013). TP53-independent Function of miR-34a via HDAC1 and p21CIP1/WAF1. Mol. Ther..

[75] Huang R., Zhou P.K. (2021). DNA damage repair: Historical perspectives, mechanistic pathways and clinical translation for targeted cancer therapy. Signal Transduct. Target. Ther..

