# Supplementary material for: A Boolean Model of the Proliferative Role of the lncRNA XIST in Non-Small Cell Lung Cancer Cells
Source: Biology (Basel). 2022 Mar 22;11(4):480. doi: 10.3390/biology11040480 (PMC9024590; doi:10.3390/biology11040480)
Supplement: Supplementary file 1 [file biology-11-00480-s001.zip › Data File S1.pdf]

A Boolean model of the proliferative role of the lncRNA XIST in non-small cell lung cancer cells

Data File S1

Shantanu Gupta [1,‡] *, Daner A. Silveira [2,‡], Ronaldo F. Hashimoto [1,‡] and Jose Carlos M. Mombach [2,‡*]

[1] Instituto de Matemática e Estatística, Departamento de Ciência da Computação, Universidade de São Paulo, Rua do Matão 1010, 05508-090, São Paulo - SP, Brasil
[2] Departamento de Física, Universidade Federal de Santa Maria, RS, Brazil

[‡]These authors contributed equally to this work

Correspondence: shantanu.gupta@ime.usp.br; +55-11-30916135:
jcmombach@ufsm.br; +55-55-32209521 (J.C.M.M)

Code of the model in .ginml file format used for the GINsim 3.0.0b software.

```
<?xml version="1.0" encoding="UTF-8"?>
<!DOCTYPE gxl SYSTEM "http://gin.univ-mrs.fr/GINsim/GINML_2_1.dtd">
<gxl>
  <graph id="defaultID" class="regulatory" nodeorder="DNA_Damage ATM Mdm2 p53 Wip1 p53_A p53_K
        miR_34a miR_16 miR_449a lnc_XIST p21 p53INP1 c_Myc YY1 C_Met Cdc25A CDK46_CycD
       CDK2_CycE RB E2F1 Sirt_1 HDAC1 BMI1 PUMA BCL2 BAX Caspase3 Poliferation Senescence
       Apoptosis">
    <node id="DNA_Damage" maxvalue="1">
      <parameter val="1" idActiveInteractions="DNA_Damage:DNA_Damage:1"/>
      <annotation>
        <comment>DNA_Damage is an input node marked with an auto-regulation.</comment>
      </annotation>
    </node>
    <node id="ATM" maxvalue="1">
      <parameter val="1" idActiveInteractions="DNA_Damage:ATM:1"/>
      <parameter val="1" idActiveInteractions="DNA_Damage:ATM:1 HDAC1:ATM:1"/>
      <parameter val="1" idActiveInteractions="DNA_Damage:ATM:1 E2F1:ATM:1"/>
      <parameter val="1" idActiveInteractions="DNA_Damage:ATM:1 E2F1:ATM:1 HDAC1:ATM:1"/>
      <parameter val="1" idActiveInteractions="DNA_Damage:ATM:1 Wip1:ATM:1"/>
      <parameter val="1" idActiveInteractions="DNA_Damage:ATM:1 Wip1:ATM:1 E2F1:ATM:1"/>
      <parameter val="1" idActiveInteractions="DNA_Damage:ATM:1 Wip1:ATM:1 E2F1:ATM:1
          HDAC1:ATM:1"/>
    </node>
    <node id="Mdm2" maxvalue="1">
      <parameter val="1"/>
      <parameter val="1" idActiveInteractions="YY1:Mdm2:1"/>
      <parameter val="1" idActiveInteractions="Wip1:Mdm2:1 YY1:Mdm2:1"/>
      <parameter val="1" idActiveInteractions="p53:Mdm2:1"/>
      <parameter val="1" idActiveInteractions="p53:Mdm2:1 YY1:Mdm2:1"/>
      <parameter val="1" idActiveInteractions="p53:Mdm2:1 Wip1:Mdm2:1"/>
      <parameter val="1" idActiveInteractions="p53:Mdm2:1 Wip1:Mdm2:1 YY1:Mdm2:1"/>
    </node>
    <node id="p53" maxvalue="1">
      <parameter val="1"/>
      <parameter val="1" idActiveInteractions="ATM:p53:1"/>
      <parameter val="1" idActiveInteractions="ATM:p53:1 Sirt_1:p53:1"/>
    </node>
    <node id="Wip1" maxvalue="1">
      <parameter val="1" idActiveInteractions="p53_A:Wip1:1"/>
    </node>
    <node id="p53_A" maxvalue="1">
      <parameter val="1"/>
      <parameter val="1" idActiveInteractions="p53:p53_A:1"/>
      <parameter val="1" idActiveInteractions="p53:p53_A:1 p53INP1:p53_A:1"/>
    </node>
    <node id="p53_K" maxvalue="1">
      <parameter val="1" idActiveInteractions="p53:p53_K:1"/>
      <parameter val="1" idActiveInteractions="p53:p53_K:1 Sirt_1:p53_K:1"/>
      <parameter val="1" idActiveInteractions="p53:p53_K:1 Wip1:p53_K:1"/>
    </node>
    <node id="miR_34a" maxvalue="1">
      <parameter val="1" idActiveInteractions="p53:miR_34a:1"/>
      <parameter val="1" idActiveInteractions="ATM:miR_34a:1"/>
      <parameter val="1" idActiveInteractions="ATM:miR_34a:1 p53:miR_34a:1"/>
    </node>
    <node id="miR_16" maxvalue="1">
      <parameter val="1" idActiveInteractions="p53:miR_16:1"/>
      <parameter val="1" idActiveInteractions="ATM:miR_16:1"/>
```

```
55        <parameter val="1" idActiveInteractions="ATM:miR_16:1 p53:miR_16:1"/>
56      </node>
57      <node id="miR_449a" maxvalue="1">
58        <parameter val="1" idActiveInteractions="DNA_Damage:miR_449a:1"/>
59      </node>
60      <node id="lnc_XIST" maxvalue="1">
61        <parameter val="1" idActiveInteractions="YY1:lnc_XIST:1"/>
62        <parameter val="1" idActiveInteractions="miR_449a:lnc_XIST:1 YY1:lnc_XIST:1"/>
63        <parameter val="1" idActiveInteractions="miR_16:lnc_XIST:1 YY1:lnc_XIST:1"/>
64        <parameter val="1" idActiveInteractions="miR_16:lnc_XIST:1 miR_449a:lnc_XIST:1
              YY1:lnc_XIST:1"/>
65        <parameter val="1" idActiveInteractions="miR_34a:lnc_XIST:1 YY1:lnc_XIST:1"/>
66        <parameter val="1" idActiveInteractions="miR_34a:lnc_XIST:1 miR_449a:lnc_XIST:1
              YY1:lnc_XIST:1"/>
67        <parameter val="1" idActiveInteractions="miR_34a:lnc_XIST:1 miR_16:lnc_XIST:1
              YY1:lnc_XIST:1"/>
68      </node>
69      <node id="p21" maxvalue="1">
70        <parameter val="1"/>
71        <parameter val="1" idActiveInteractions="BMI1:p21:1"/>
72        <parameter val="1" idActiveInteractions="HDAC1:p21:1"/>
73        <parameter val="1" idActiveInteractions="HDAC1:p21:1 BMI1:p21:1"/>
74        <parameter val="1" idActiveInteractions="YY1:p21:1"/>
75        <parameter val="1" idActiveInteractions="YY1:p21:1 BMI1:p21:1"/>
76        <parameter val="1" idActiveInteractions="YY1:p21:1 HDAC1:p21:1"/>
77        <parameter val="1" idActiveInteractions="YY1:p21:1 HDAC1:p21:1 BMI1:p21:1"/>
78        <parameter val="1" idActiveInteractions="c_Myc:p21:1"/>
79        <parameter val="1" idActiveInteractions="c_Myc:p21:1 BMI1:p21:1"/>
80        <parameter val="1" idActiveInteractions="c_Myc:p21:1 HDAC1:p21:1"/>
81        <parameter val="1" idActiveInteractions="c_Myc:p21:1 HDAC1:p21:1 BMI1:p21:1"/>
82        <parameter val="1" idActiveInteractions="c_Myc:p21:1 YY1:p21:1"/>
83        <parameter val="1" idActiveInteractions="c_Myc:p21:1 YY1:p21:1 BMI1:p21:1"/>
84        <parameter val="1" idActiveInteractions="c_Myc:p21:1 YY1:p21:1 HDAC1:p21:1"/>
85        <parameter val="1" idActiveInteractions="c_Myc:p21:1 YY1:p21:1 HDAC1:p21:1 BMI1:p21:1"/>
86        <parameter val="1" idActiveInteractions="lnc_XIST:p21:1"/>
87        <parameter val="1" idActiveInteractions="lnc_XIST:p21:1 BMI1:p21:1"/>
88        <parameter val="1" idActiveInteractions="lnc_XIST:p21:1 HDAC1:p21:1"/>
89        <parameter val="1" idActiveInteractions="lnc_XIST:p21:1 HDAC1:p21:1 BMI1:p21:1"/>
90        <parameter val="1" idActiveInteractions="lnc_XIST:p21:1 YY1:p21:1"/>
91        <parameter val="1" idActiveInteractions="lnc_XIST:p21:1 YY1:p21:1 BMI1:p21:1"/>
92        <parameter val="1" idActiveInteractions="lnc_XIST:p21:1 YY1:p21:1 HDAC1:p21:1"/>
93        <parameter val="1" idActiveInteractions="lnc_XIST:p21:1 YY1:p21:1 HDAC1:p21:1 BMI1:p21:1
              "/>
94        <parameter val="1" idActiveInteractions="lnc_XIST:p21:1 c_Myc:p21:1"/>
95        <parameter val="1" idActiveInteractions="lnc_XIST:p21:1 c_Myc:p21:1 BMI1:p21:1"/>
96        <parameter val="1" idActiveInteractions="lnc_XIST:p21:1 c_Myc:p21:1 HDAC1:p21:1"/>
97        <parameter val="1" idActiveInteractions="lnc_XIST:p21:1 c_Myc:p21:1 HDAC1:p21:1
              BMI1:p21:1"/>
98        <parameter val="1" idActiveInteractions="lnc_XIST:p21:1 c_Myc:p21:1 YY1:p21:1"/>
99        <parameter val="1" idActiveInteractions="lnc_XIST:p21:1 c_Myc:p21:1 YY1:p21:1 BMI1:p21:1
              "/>
100       <parameter val="1" idActiveInteractions="lnc_XIST:p21:1 c_Myc:p21:1 YY1:p21:1
              HDAC1:p21:1"/>
101       <parameter val="1" idActiveInteractions="p53_A:p21:1"/>
102       <parameter val="1" idActiveInteractions="p53_A:p21:1 Caspase3:p21:1"/>
103       <parameter val="1" idActiveInteractions="p53_A:p21:1 BMI1:p21:1"/>
104       <parameter val="1" idActiveInteractions="p53_A:p21:1 BMI1:p21:1 Caspase3:p21:1"/>
105       <parameter val="1" idActiveInteractions="p53_A:p21:1 HDAC1:p21:1"/>
106       <parameter val="1" idActiveInteractions="p53_A:p21:1 HDAC1:p21:1 Caspase3:p21:1"/>
107       <parameter val="1" idActiveInteractions="p53_A:p21:1 HDAC1:p21:1 BMI1:p21:1"/>
108       <parameter val="1" idActiveInteractions="p53_A:p21:1 HDAC1:p21:1 BMI1:p21:1
              Caspase3:p21:1"/>
```

```
109     <parameter val="1" idActiveInteractions="p53_A:p21:1 YY1:p21:1"/>
110     <parameter val="1" idActiveInteractions="p53_A:p21:1 YY1:p21:1 Caspase3:p21:1"/>
111     <parameter val="1" idActiveInteractions="p53_A:p21:1 YY1:p21:1 BMI1:p21:1"/>
112     <parameter val="1" idActiveInteractions="p53_A:p21:1 YY1:p21:1 BMI1:p21:1 Caspase3:p21:1
            "/>
113     <parameter val="1" idActiveInteractions="p53_A:p21:1 YY1:p21:1 HDAC1:p21:1"/>
114     <parameter val="1" idActiveInteractions="p53_A:p21:1 YY1:p21:1 HDAC1:p21:1
            Caspase3:p21:1"/>
115     <parameter val="1" idActiveInteractions="p53_A:p21:1 YY1:p21:1 HDAC1:p21:1 BMI1:p21:1"/>
116     <parameter val="1" idActiveInteractions="p53_A:p21:1 YY1:p21:1 HDAC1:p21:1 BMI1:p21:1
            Caspase3:p21:1"/>
117     <parameter val="1" idActiveInteractions="p53_A:p21:1 c_Myc:p21:1"/>
118     <parameter val="1" idActiveInteractions="p53_A:p21:1 c_Myc:p21:1 Caspase3:p21:1"/>
119     <parameter val="1" idActiveInteractions="p53_A:p21:1 c_Myc:p21:1 BMI1:p21:1"/>
120     <parameter val="1" idActiveInteractions="p53_A:p21:1 c_Myc:p21:1 BMI1:p21:1
            Caspase3:p21:1"/>
121     <parameter val="1" idActiveInteractions="p53_A:p21:1 c_Myc:p21:1 HDAC1:p21:1"/>
122     <parameter val="1" idActiveInteractions="p53_A:p21:1 c_Myc:p21:1 HDAC1:p21:1
            Caspase3:p21:1"/>
123     <parameter val="1" idActiveInteractions="p53_A:p21:1 c_Myc:p21:1 HDAC1:p21:1 BMI1:p21:1"
            />
124     <parameter val="1" idActiveInteractions="p53_A:p21:1 c_Myc:p21:1 HDAC1:p21:1 BMI1:p21:1
            Caspase3:p21:1"/>
125     <parameter val="1" idActiveInteractions="p53_A:p21:1 c_Myc:p21:1 YY1:p21:1"/>
126     <parameter val="1" idActiveInteractions="p53_A:p21:1 c_Myc:p21:1 YY1:p21:1
            Caspase3:p21:1"/>
127     <parameter val="1" idActiveInteractions="p53_A:p21:1 c_Myc:p21:1 YY1:p21:1 BMI1:p21:1"/>
128     <parameter val="1" idActiveInteractions="p53_A:p21:1 c_Myc:p21:1 YY1:p21:1 BMI1:p21:1
            Caspase3:p21:1"/>
129     <parameter val="1" idActiveInteractions="p53_A:p21:1 c_Myc:p21:1 YY1:p21:1 HDAC1:p21:1"/
            >
130     <parameter val="1" idActiveInteractions="p53_A:p21:1 c_Myc:p21:1 YY1:p21:1 HDAC1:p21:1
            Caspase3:p21:1"/>
131     <parameter val="1" idActiveInteractions="p53_A:p21:1 c_Myc:p21:1 YY1:p21:1 HDAC1:p21:1
            BMI1:p21:1"/>
132     <parameter val="1" idActiveInteractions="p53_A:p21:1 c_Myc:p21:1 YY1:p21:1 HDAC1:p21:1
            BMI1:p21:1 Caspase3:p21:1"/>
133     <parameter val="1" idActiveInteractions="p53_A:p21:1 lnc_XIST:p21:1"/>
134     <parameter val="1" idActiveInteractions="p53_A:p21:1 lnc_XIST:p21:1 Caspase3:p21:1"/>
135     <parameter val="1" idActiveInteractions="p53_A:p21:1 lnc_XIST:p21:1 BMI1:p21:1"/>
136     <parameter val="1" idActiveInteractions="p53_A:p21:1 lnc_XIST:p21:1 BMI1:p21:1
            Caspase3:p21:1"/>
137     <parameter val="1" idActiveInteractions="p53_A:p21:1 lnc_XIST:p21:1 HDAC1:p21:1"/>
138     <parameter val="1" idActiveInteractions="p53_A:p21:1 lnc_XIST:p21:1 HDAC1:p21:1
            Caspase3:p21:1"/>
139     <parameter val="1" idActiveInteractions="p53_A:p21:1 lnc_XIST:p21:1 HDAC1:p21:1
            BMI1:p21:1"/>
140     <parameter val="1" idActiveInteractions="p53_A:p21:1 lnc_XIST:p21:1 HDAC1:p21:1
            BMI1:p21:1 Caspase3:p21:1"/>
141     <parameter val="1" idActiveInteractions="p53_A:p21:1 lnc_XIST:p21:1 YY1:p21:1"/>
142     <parameter val="1" idActiveInteractions="p53_A:p21:1 lnc_XIST:p21:1 YY1:p21:1
            Caspase3:p21:1"/>
143     <parameter val="1" idActiveInteractions="p53_A:p21:1 lnc_XIST:p21:1 YY1:p21:1 BMI1:p21:1
            "/>
144     <parameter val="1" idActiveInteractions="p53_A:p21:1 lnc_XIST:p21:1 YY1:p21:1 BMI1:p21:1
             Caspase3:p21:1"/>
145     <parameter val="1" idActiveInteractions="p53_A:p21:1 lnc_XIST:p21:1 YY1:p21:1
            HDAC1:p21:1"/>
146     <parameter val="1" idActiveInteractions="p53_A:p21:1 lnc_XIST:p21:1 YY1:p21:1
            HDAC1:p21:1 Caspase3:p21:1"/>
147     <parameter val="1" idActiveInteractions="p53_A:p21:1 lnc_XIST:p21:1 YY1:p21:1
            HDAC1:p21:1 BMI1:p21:1"/>
```

```
148       <parameter val="1" idActiveInteractions="p53_A:p21:1 lnc_XIST:p21:1 YY1:p21:1
              HDAC1:p21:1 BMI1:p21:1 Caspase3:p21:1"/>
149       <parameter val="1" idActiveInteractions="p53_A:p21:1 lnc_XIST:p21:1 c_Myc:p21:1"/>
150       <parameter val="1" idActiveInteractions="p53_A:p21:1 lnc_XIST:p21:1 c_Myc:p21:1
              Caspase3:p21:1"/>
151       <parameter val="1" idActiveInteractions="p53_A:p21:1 lnc_XIST:p21:1 c_Myc:p21:1
              BMI1:p21:1"/>
152       <parameter val="1" idActiveInteractions="p53_A:p21:1 lnc_XIST:p21:1 c_Myc:p21:1
              BMI1:p21:1 Caspase3:p21:1"/>
153       <parameter val="1" idActiveInteractions="p53_A:p21:1 lnc_XIST:p21:1 c_Myc:p21:1
              HDAC1:p21:1"/>
154       <parameter val="1" idActiveInteractions="p53_A:p21:1 lnc_XIST:p21:1 c_Myc:p21:1
              HDAC1:p21:1 Caspase3:p21:1"/>
155       <parameter val="1" idActiveInteractions="p53_A:p21:1 lnc_XIST:p21:1 c_Myc:p21:1
              HDAC1:p21:1 BMI1:p21:1"/>
156       <parameter val="1" idActiveInteractions="p53_A:p21:1 lnc_XIST:p21:1 c_Myc:p21:1
              HDAC1:p21:1 BMI1:p21:1 Caspase3:p21:1"/>
157       <parameter val="1" idActiveInteractions="p53_A:p21:1 lnc_XIST:p21:1 c_Myc:p21:1
              YY1:p21:1"/>
158       <parameter val="1" idActiveInteractions="p53_A:p21:1 lnc_XIST:p21:1 c_Myc:p21:1
              YY1:p21:1 Caspase3:p21:1"/>
159       <parameter val="1" idActiveInteractions="p53_A:p21:1 lnc_XIST:p21:1 c_Myc:p21:1
              YY1:p21:1 BMI1:p21:1"/>
160       <parameter val="1" idActiveInteractions="p53_A:p21:1 lnc_XIST:p21:1 c_Myc:p21:1
              YY1:p21:1 BMI1:p21:1 Caspase3:p21:1"/>
161       <parameter val="1" idActiveInteractions="p53_A:p21:1 lnc_XIST:p21:1 c_Myc:p21:1
              YY1:p21:1 HDAC1:p21:1"/>
162       <parameter val="1" idActiveInteractions="p53_A:p21:1 lnc_XIST:p21:1 c_Myc:p21:1
              YY1:p21:1 HDAC1:p21:1 Caspase3:p21:1"/>
163       <parameter val="1" idActiveInteractions="p53_A:p21:1 lnc_XIST:p21:1 c_Myc:p21:1
              YY1:p21:1 HDAC1:p21:1 BMI1:p21:1"/>
164       <parameter val="1" idActiveInteractions="p53_A:p21:1 lnc_XIST:p21:1 c_Myc:p21:1
              YY1:p21:1 HDAC1:p21:1 BMI1:p21:1 Caspase3:p21:1"/>
165     </node>
166     <node id="p53INP1" maxvalue="1">
167       <parameter val="1" idActiveInteractions="p53_K:p53INP1:1"/>
168       <parameter val="1" idActiveInteractions="p53_A:p53INP1:1"/>
169       <parameter val="1" idActiveInteractions="p53_A:p53INP1:1 p53_K:p53INP1:1"/>
170     </node>
171     <node id="c_Myc" maxvalue="1">
172       <parameter val="1"/>
173       <parameter val="1" idActiveInteractions="E2F1:c_Myc:1"/>
174       <parameter val="1" idActiveInteractions="C_Met:c_Myc:1"/>
175       <parameter val="1" idActiveInteractions="C_Met:c_Myc:1 E2F1:c_Myc:1"/>
176       <parameter val="1" idActiveInteractions="miR_449a:c_Myc:1"/>
177       <parameter val="1" idActiveInteractions="miR_449a:c_Myc:1 E2F1:c_Myc:1"/>
178       <parameter val="1" idActiveInteractions="miR_449a:c_Myc:1 C_Met:c_Myc:1"/>
179       <parameter val="1" idActiveInteractions="miR_449a:c_Myc:1 C_Met:c_Myc:1 E2F1:c_Myc:1"/>
180       <parameter val="1" idActiveInteractions="miR_34a:c_Myc:1"/>
181       <parameter val="1" idActiveInteractions="miR_34a:c_Myc:1 E2F1:c_Myc:1"/>
182       <parameter val="1" idActiveInteractions="miR_34a:c_Myc:1 C_Met:c_Myc:1"/>
183       <parameter val="1" idActiveInteractions="miR_34a:c_Myc:1 C_Met:c_Myc:1 E2F1:c_Myc:1"/>
184       <parameter val="1" idActiveInteractions="miR_34a:c_Myc:1 miR_449a:c_Myc:1 E2F1:c_Myc:1"/
              >
185       <parameter val="1" idActiveInteractions="miR_34a:c_Myc:1 miR_449a:c_Myc:1 C_Met:c_Myc:1"
              />
186       <parameter val="1" idActiveInteractions="miR_34a:c_Myc:1 miR_449a:c_Myc:1 C_Met:c_Myc:1
              E2F1:c_Myc:1"/>
187     </node>
188     <node id="YY1" maxvalue="1">
189       <parameter val="1" idActiveInteractions="c_Myc:YY1:1"/>
190       <parameter val="1" idActiveInteractions="c_Myc:YY1:1 RB:YY1:1"/>
```

```
      <parameter val="1" idActiveInteractions="miR_34a:YY1:1 c_Myc:YY1:1"/>
    </node>
    <node id="C_Met" maxvalue="1">
      <parameter val="1" idActiveInteractions="c_Myc:C_Met:1"/>
    </node>
    <node id="Cdc25A" maxvalue="1">
      <parameter val="1"/>
    </node>
    <node id="CDK46_CycD" maxvalue="1">
      <parameter val="1" idActiveInteractions="Cdc25A:CDK46_CycD:1"/>
    </node>
    <node id="CDK2_CycE" maxvalue="1">
      <parameter val="1" idActiveInteractions="Cdc25A:CDK2_CycE:1 E2F1:CDK2_CycE:1"/>
    </node>
    <node id="RB" maxvalue="1">
      <parameter val="1"/>
    </node>
    <node id="E2F1" maxvalue="1">
      <parameter val="1"/>
      <parameter val="1" idActiveInteractions="Sirt_1:E2F1:1"/>
      <parameter val="1" idActiveInteractions="Cdc25A:E2F1:1"/>
      <parameter val="1" idActiveInteractions="Cdc25A:E2F1:1 Sirt_1:E2F1:1"/>
      <parameter val="1" idActiveInteractions="miR_449a:E2F1:1"/>
      <parameter val="1" idActiveInteractions="miR_449a:E2F1:1 Sirt_1:E2F1:1"/>
      <parameter val="1" idActiveInteractions="miR_449a:E2F1:1 Cdc25A:E2F1:1"/>
      <parameter val="1" idActiveInteractions="miR_449a:E2F1:1 Cdc25A:E2F1:1 Sirt_1:E2F1:1"/>
      <parameter val="1" idActiveInteractions="c_Myc:E2F1:1"/>
      <parameter val="1" idActiveInteractions="c_Myc:E2F1:1 Sirt_1:E2F1:1"/>
      <parameter val="1" idActiveInteractions="c_Myc:E2F1:1 RB:E2F1:1"/>
      <parameter val="1" idActiveInteractions="c_Myc:E2F1:1 RB:E2F1:1 Sirt_1:E2F1:1"/>
      <parameter val="1" idActiveInteractions="c_Myc:E2F1:1 Cdc25A:E2F1:1"/>
      <parameter val="1" idActiveInteractions="c_Myc:E2F1:1 Cdc25A:E2F1:1 Sirt_1:E2F1:1"/>
      <parameter val="1" idActiveInteractions="c_Myc:E2F1:1 Cdc25A:E2F1:1 RB:E2F1:1"/>
      <parameter val="1" idActiveInteractions="c_Myc:E2F1:1 Cdc25A:E2F1:1 RB:E2F1:1
          Sirt_1:E2F1:1"/>
      <parameter val="1" idActiveInteractions="miR_449a:E2F1:1 c_Myc:E2F1:1"/>
      <parameter val="1" idActiveInteractions="miR_449a:E2F1:1 c_Myc:E2F1:1 Sirt_1:E2F1:1"/>
      <parameter val="1" idActiveInteractions="miR_449a:E2F1:1 c_Myc:E2F1:1 RB:E2F1:1"/>
      <parameter val="1" idActiveInteractions="miR_449a:E2F1:1 c_Myc:E2F1:1 RB:E2F1:1
          Sirt_1:E2F1:1"/>
      <parameter val="1" idActiveInteractions="miR_449a:E2F1:1 c_Myc:E2F1:1 Cdc25A:E2F1:1"/>
      <parameter val="1" idActiveInteractions="miR_449a:E2F1:1 c_Myc:E2F1:1 Cdc25A:E2F1:1
          Sirt_1:E2F1:1"/>
      <parameter val="1" idActiveInteractions="miR_449a:E2F1:1 c_Myc:E2F1:1 Cdc25A:E2F1:1
          RB:E2F1:1"/>
      <parameter val="1" idActiveInteractions="miR_449a:E2F1:1 c_Myc:E2F1:1 Cdc25A:E2F1:1
          RB:E2F1:1 Sirt_1:E2F1:1"/>
      <parameter val="1" idActiveInteractions="miR_34a:E2F1:1"/>
      <parameter val="1" idActiveInteractions="miR_34a:E2F1:1 Sirt_1:E2F1:1"/>
      <parameter val="1" idActiveInteractions="miR_34a:E2F1:1 Cdc25A:E2F1:1"/>
      <parameter val="1" idActiveInteractions="miR_34a:E2F1:1 Cdc25A:E2F1:1 Sirt_1:E2F1:1"/>
      <parameter val="1" idActiveInteractions="miR_34a:E2F1:1 c_Myc:E2F1:1"/>
      <parameter val="1" idActiveInteractions="miR_34a:E2F1:1 c_Myc:E2F1:1 Sirt_1:E2F1:1"/>
      <parameter val="1" idActiveInteractions="miR_34a:E2F1:1 c_Myc:E2F1:1 RB:E2F1:1"/>
      <parameter val="1" idActiveInteractions="miR_34a:E2F1:1 c_Myc:E2F1:1 RB:E2F1:1
          Sirt_1:E2F1:1"/>
      <parameter val="1" idActiveInteractions="miR_34a:E2F1:1 c_Myc:E2F1:1 Cdc25A:E2F1:1"/>
      <parameter val="1" idActiveInteractions="miR_34a:E2F1:1 c_Myc:E2F1:1 Cdc25A:E2F1:1
          Sirt_1:E2F1:1"/>
      <parameter val="1" idActiveInteractions="miR_34a:E2F1:1 c_Myc:E2F1:1 Cdc25A:E2F1:1
          RB:E2F1:1"/>
      <parameter val="1" idActiveInteractions="miR_34a:E2F1:1 c_Myc:E2F1:1 Cdc25A:E2F1:1
```

```
        RB:E2F1:1 Sirt_1:E2F1:1"/>
    <parameter val="1" idActiveInteractions="miR_34a:E2F1:1 miR_449a:E2F1:1"/>
    <parameter val="1" idActiveInteractions="miR_34a:E2F1:1 miR_449a:E2F1:1 Cdc25A:E2F1:1"/>
    <parameter val="1" idActiveInteractions="miR_34a:E2F1:1 miR_449a:E2F1:1 c_Myc:E2F1:1"/>
    <parameter val="1" idActiveInteractions="miR_34a:E2F1:1 miR_449a:E2F1:1 c_Myc:E2F1:1
        Sirt_1:E2F1:1"/>
    <parameter val="1" idActiveInteractions="miR_34a:E2F1:1 miR_449a:E2F1:1 c_Myc:E2F1:1
        RB:E2F1:1"/>
    <parameter val="1" idActiveInteractions="miR_34a:E2F1:1 miR_449a:E2F1:1 c_Myc:E2F1:1
        RB:E2F1:1 Sirt_1:E2F1:1"/>
    <parameter val="1" idActiveInteractions="miR_34a:E2F1:1 miR_449a:E2F1:1 c_Myc:E2F1:1
        Cdc25A:E2F1:1"/>
    <parameter val="1" idActiveInteractions="miR_34a:E2F1:1 miR_449a:E2F1:1 c_Myc:E2F1:1
        Cdc25A:E2F1:1 Sirt_1:E2F1:1"/>
    <parameter val="1" idActiveInteractions="miR_34a:E2F1:1 miR_449a:E2F1:1 c_Myc:E2F1:1
        Cdc25A:E2F1:1 RB:E2F1:1"/>
    <parameter val="1" idActiveInteractions="miR_34a:E2F1:1 miR_449a:E2F1:1 c_Myc:E2F1:1
        Cdc25A:E2F1:1 RB:E2F1:1 Sirt_1:E2F1:1"/>
    <parameter val="1" idActiveInteractions="ATM:E2F1:1"/>
    <parameter val="1" idActiveInteractions="ATM:E2F1:1 Sirt_1:E2F1:1"/>
    <parameter val="1" idActiveInteractions="ATM:E2F1:1 Cdc25A:E2F1:1"/>
    <parameter val="1" idActiveInteractions="ATM:E2F1:1 Cdc25A:E2F1:1 Sirt_1:E2F1:1"/>
    <parameter val="1" idActiveInteractions="ATM:E2F1:1 miR_449a:E2F1:1"/>
    <parameter val="1" idActiveInteractions="ATM:E2F1:1 miR_449a:E2F1:1 Sirt_1:E2F1:1"/>
    <parameter val="1" idActiveInteractions="ATM:E2F1:1 miR_449a:E2F1:1 Cdc25A:E2F1:1"/>
    <parameter val="1" idActiveInteractions="ATM:E2F1:1 miR_449a:E2F1:1 Cdc25A:E2F1:1
        Sirt_1:E2F1:1"/>
    <parameter val="1" idActiveInteractions="ATM:E2F1:1 c_Myc:E2F1:1"/>
    <parameter val="1" idActiveInteractions="ATM:E2F1:1 c_Myc:E2F1:1 Sirt_1:E2F1:1"/>
    <parameter val="1" idActiveInteractions="ATM:E2F1:1 c_Myc:E2F1:1 RB:E2F1:1"/>
    <parameter val="1" idActiveInteractions="ATM:E2F1:1 c_Myc:E2F1:1 RB:E2F1:1 Sirt_1:E2F1:1
        "/>
    <parameter val="1" idActiveInteractions="ATM:E2F1:1 c_Myc:E2F1:1 Cdc25A:E2F1:1"/>
    <parameter val="1" idActiveInteractions="ATM:E2F1:1 c_Myc:E2F1:1 Cdc25A:E2F1:1
        Sirt_1:E2F1:1"/>
    <parameter val="1" idActiveInteractions="ATM:E2F1:1 c_Myc:E2F1:1 Cdc25A:E2F1:1 RB:E2F1:1
        "/>
    <parameter val="1" idActiveInteractions="ATM:E2F1:1 c_Myc:E2F1:1 Cdc25A:E2F1:1 RB:E2F1:1
         Sirt_1:E2F1:1"/>
    <parameter val="1" idActiveInteractions="ATM:E2F1:1 miR_449a:E2F1:1 c_Myc:E2F1:1"/>
    <parameter val="1" idActiveInteractions="ATM:E2F1:1 miR_449a:E2F1:1 c_Myc:E2F1:1
        Sirt_1:E2F1:1"/>
    <parameter val="1" idActiveInteractions="ATM:E2F1:1 miR_449a:E2F1:1 c_Myc:E2F1:1
        RB:E2F1:1"/>
    <parameter val="1" idActiveInteractions="ATM:E2F1:1 miR_449a:E2F1:1 c_Myc:E2F1:1
        RB:E2F1:1 Sirt_1:E2F1:1"/>
    <parameter val="1" idActiveInteractions="ATM:E2F1:1 miR_449a:E2F1:1 c_Myc:E2F1:1
        Cdc25A:E2F1:1"/>
    <parameter val="1" idActiveInteractions="ATM:E2F1:1 miR_449a:E2F1:1 c_Myc:E2F1:1
        Cdc25A:E2F1:1 Sirt_1:E2F1:1"/>
    <parameter val="1" idActiveInteractions="ATM:E2F1:1 miR_449a:E2F1:1 c_Myc:E2F1:1
        Cdc25A:E2F1:1 RB:E2F1:1"/>
    <parameter val="1" idActiveInteractions="ATM:E2F1:1 miR_449a:E2F1:1 c_Myc:E2F1:1
        Cdc25A:E2F1:1 RB:E2F1:1 Sirt_1:E2F1:1"/>
    <parameter val="1" idActiveInteractions="ATM:E2F1:1 miR_34a:E2F1:1"/>
    <parameter val="1" idActiveInteractions="ATM:E2F1:1 miR_34a:E2F1:1 Sirt_1:E2F1:1"/>
    <parameter val="1" idActiveInteractions="ATM:E2F1:1 miR_34a:E2F1:1 Cdc25A:E2F1:1"/>
    <parameter val="1" idActiveInteractions="ATM:E2F1:1 miR_34a:E2F1:1 Cdc25A:E2F1:1
        Sirt_1:E2F1:1"/>
    <parameter val="1" idActiveInteractions="ATM:E2F1:1 miR_34a:E2F1:1 c_Myc:E2F1:1"/>
    <parameter val="1" idActiveInteractions="ATM:E2F1:1 miR_34a:E2F1:1 c_Myc:E2F1:1
        Sirt_1:E2F1:1"/>
```

```
285       <parameter val="1" idActiveInteractions="ATM:E2F1:1 miR_34a:E2F1:1 c_Myc:E2F1:1
              RB:E2F1:1"/>
286       <parameter val="1" idActiveInteractions="ATM:E2F1:1 miR_34a:E2F1:1 c_Myc:E2F1:1
              RB:E2F1:1 Sirt_1:E2F1:1"/>
287       <parameter val="1" idActiveInteractions="ATM:E2F1:1 miR_34a:E2F1:1 c_Myc:E2F1:1
              Cdc25A:E2F1:1"/>
288       <parameter val="1" idActiveInteractions="ATM:E2F1:1 miR_34a:E2F1:1 c_Myc:E2F1:1
              Cdc25A:E2F1:1 Sirt_1:E2F1:1"/>
289       <parameter val="1" idActiveInteractions="ATM:E2F1:1 miR_34a:E2F1:1 c_Myc:E2F1:1
              Cdc25A:E2F1:1 RB:E2F1:1"/>
290       <parameter val="1" idActiveInteractions="ATM:E2F1:1 miR_34a:E2F1:1 c_Myc:E2F1:1
              Cdc25A:E2F1:1 RB:E2F1:1 Sirt_1:E2F1:1"/>
291       <parameter val="1" idActiveInteractions="ATM:E2F1:1 miR_34a:E2F1:1 miR_449a:E2F1:1"/>
292       <parameter val="1" idActiveInteractions="ATM:E2F1:1 miR_34a:E2F1:1 miR_449a:E2F1:1
              Cdc25A:E2F1:1"/>
293       <parameter val="1" idActiveInteractions="ATM:E2F1:1 miR_34a:E2F1:1 miR_449a:E2F1:1
              Cdc25A:E2F1:1 Sirt_1:E2F1:1"/>
294       <parameter val="1" idActiveInteractions="ATM:E2F1:1 miR_34a:E2F1:1 miR_449a:E2F1:1
              c_Myc:E2F1:1"/>
295       <parameter val="1" idActiveInteractions="ATM:E2F1:1 miR_34a:E2F1:1 miR_449a:E2F1:1
              c_Myc:E2F1:1 Sirt_1:E2F1:1"/>
296       <parameter val="1" idActiveInteractions="ATM:E2F1:1 miR_34a:E2F1:1 miR_449a:E2F1:1
              c_Myc:E2F1:1 RB:E2F1:1"/>
297       <parameter val="1" idActiveInteractions="ATM:E2F1:1 miR_34a:E2F1:1 miR_449a:E2F1:1
              c_Myc:E2F1:1 RB:E2F1:1 Sirt_1:E2F1:1"/>
298       <parameter val="1" idActiveInteractions="ATM:E2F1:1 miR_34a:E2F1:1 miR_449a:E2F1:1
              c_Myc:E2F1:1 Cdc25A:E2F1:1"/>
299       <parameter val="1" idActiveInteractions="ATM:E2F1:1 miR_34a:E2F1:1 miR_449a:E2F1:1
              c_Myc:E2F1:1 Cdc25A:E2F1:1 Sirt_1:E2F1:1"/>
300       <parameter val="1" idActiveInteractions="ATM:E2F1:1 miR_34a:E2F1:1 miR_449a:E2F1:1
              c_Myc:E2F1:1 Cdc25A:E2F1:1 RB:E2F1:1"/>
301       <parameter val="1" idActiveInteractions="ATM:E2F1:1 miR_34a:E2F1:1 miR_449a:E2F1:1
              c_Myc:E2F1:1 Cdc25A:E2F1:1 RB:E2F1:1 Sirt_1:E2F1:1"/>
302     </node>
303     <node id="Sirt_1" maxvalue="1">
304       <parameter val="1"/>
305       <parameter val="1" idActiveInteractions="E2F1:Sirt_1:1"/>
306       <parameter val="1" idActiveInteractions="E2F1:Sirt_1:1 HDAC1:Sirt_1:1"/>
307       <parameter val="1" idActiveInteractions="miR_449a:Sirt_1:1 E2F1:Sirt_1:1"/>
308       <parameter val="1" idActiveInteractions="miR_449a:Sirt_1:1 E2F1:Sirt_1:1 HDAC1:Sirt_1:1"
              />
309       <parameter val="1" idActiveInteractions="miR_34a:Sirt_1:1 E2F1:Sirt_1:1"/>
310       <parameter val="1" idActiveInteractions="miR_34a:Sirt_1:1 E2F1:Sirt_1:1 HDAC1:Sirt_1:1"/
              >
311       <parameter val="1" idActiveInteractions="miR_34a:Sirt_1:1 miR_449a:Sirt_1:1
              E2F1:Sirt_1:1"/>
312       <parameter val="1" idActiveInteractions="miR_34a:Sirt_1:1 miR_449a:Sirt_1:1
              E2F1:Sirt_1:1 HDAC1:Sirt_1:1"/>
313     </node>
314     <node id="HDAC1" maxvalue="1">
315       <parameter val="1"/>
316       <parameter val="1" idActiveInteractions="Sirt_1:HDAC1:1"/>
317       <parameter val="1" idActiveInteractions="miR_449a:HDAC1:1"/>
318       <parameter val="1" idActiveInteractions="miR_449a:HDAC1:1 Sirt_1:HDAC1:1"/>
319       <parameter val="1" idActiveInteractions="miR_34a:HDAC1:1"/>
320       <parameter val="1" idActiveInteractions="miR_34a:HDAC1:1 Sirt_1:HDAC1:1"/>
321       <parameter val="1" idActiveInteractions="miR_34a:HDAC1:1 miR_449a:HDAC1:1"/>
322       <parameter val="1" idActiveInteractions="miR_34a:HDAC1:1 miR_449a:HDAC1:1 Sirt_1:HDAC1:1
              "/>
323       <parameter val="1" idActiveInteractions="DNA_Damage:HDAC1:1"/>
324       <parameter val="1" idActiveInteractions="DNA_Damage:HDAC1:1 Sirt_1:HDAC1:1"/>
325       <parameter val="1" idActiveInteractions="DNA_Damage:HDAC1:1 miR_449a:HDAC1:1"/>
```

```
      <parameter val="1" idActiveInteractions="DNA_Damage:HDAC1:1 miR_449a:HDAC1:1
          Sirt_1:HDAC1:1"/>
      <parameter val="1" idActiveInteractions="DNA_Damage:HDAC1:1 miR_34a:HDAC1:1"/>
      <parameter val="1" idActiveInteractions="DNA_Damage:HDAC1:1 miR_34a:HDAC1:1
          Sirt_1:HDAC1:1"/>
      <parameter val="1" idActiveInteractions="DNA_Damage:HDAC1:1 miR_34a:HDAC1:1
          miR_449a:HDAC1:1"/>
    </node>
    <node id="BMI1" maxvalue="1">
      <parameter val="1" idActiveInteractions="E2F1:BMI1:1"/>
      <parameter val="1" idActiveInteractions="c_Myc:BMI1:1"/>
      <parameter val="1" idActiveInteractions="c_Myc:BMI1:1 E2F1:BMI1:1"/>
    </node>
    <node id="PUMA" maxvalue="1">
      <parameter val="1" idActiveInteractions="p53_K:PUMA:1"/>
    </node>
    <node id="BCL2" maxvalue="1">
      <parameter val="1"/>
    </node>
    <node id="BAX" maxvalue="1">
      <parameter val="1" idActiveInteractions="p53_K:BAX:1"/>
    </node>
    <node id="Caspase3" maxvalue="1">
      <parameter val="1" idActiveInteractions="BAX:Caspase3:1"/>
      <parameter val="1" idActiveInteractions="BCL2:Caspase3:1 BAX:Caspase3:1"/>
      <parameter val="1" idActiveInteractions="p21:Caspase3:1 BAX:Caspase3:1"/>
    </node>
    <node id="Poliferation" maxvalue="1">
      <parameter val="1" idActiveInteractions="CDK2_CycE:Poliferation:1"/>
    </node>
    <node id="Senescence" maxvalue="1">
      <parameter val="1" idActiveInteractions="p21:Senescence:1"/>
    </node>
    <node id="Apoptosis" maxvalue="1">
      <parameter val="1" idActiveInteractions="Caspase3:Apoptosis:1"/>
    </node>
    <edge id="DNA_Damage:DNA_Damage:1" from="DNA_Damage" to="DNA_Damage" sign="positive"
        minvalue="1"/>
    <edge id="DNA_Damage:ATM:1" from="DNA_Damage" to="ATM" sign="positive" minvalue="1"/>
    <edge id="Wip1:ATM:1" from="Wip1" to="ATM" sign="negative" minvalue="1"/>
    <edge id="E2F1:ATM:1" from="E2F1" to="ATM" sign="positive" minvalue="1"/>
    <edge id="HDAC1:ATM:1" from="HDAC1" to="ATM" sign="negative" minvalue="1"/>
    <edge id="ATM:Mdm2:1" from="ATM" to="Mdm2" sign="negative" minvalue="1"/>
    <edge id="p53:Mdm2:1" from="p53" to="Mdm2" sign="positive" minvalue="1"/>
    <edge id="Wip1:Mdm2:1" from="Wip1" to="Mdm2" sign="negative" minvalue="1"/>
    <edge id="YY1:Mdm2:1" from="YY1" to="Mdm2" sign="positive" minvalue="1"/>
    <edge id="ATM:p53:1" from="ATM" to="p53" sign="positive" minvalue="1"/>
    <edge id="Mdm2:p53:1" from="Mdm2" to="p53" sign="negative" minvalue="1"/>
    <edge id="YY1:p53:1" from="YY1" to="p53" sign="negative" minvalue="1"/>
    <edge id="C_Met:p53:1" from="C_Met" to="p53" sign="negative" minvalue="1"/>
    <edge id="Sirt_1:p53:1" from="Sirt_1" to="p53" sign="negative" minvalue="1"/>
    <edge id="p53_A:Wip1:1" from="p53_A" to="Wip1" sign="positive" minvalue="1"/>
    <edge id="miR_16:Wip1:1" from="miR_16" to="Wip1" sign="negative" minvalue="1"/>
    <edge id="p53:p53_A:1" from="p53" to="p53_A" sign="positive" minvalue="1"/>
    <edge id="p53_K:p53_A:1" from="p53_K" to="p53_A" sign="negative" minvalue="1"/>
    <edge id="p53INP1:p53_A:1" from="p53INP1" to="p53_A" sign="negative" minvalue="1"/>
    <edge id="Sirt_1:p53_A:1" from="Sirt_1" to="p53_A" sign="negative" minvalue="1"/>
    <edge id="p53:p53_K:1" from="p53" to="p53_K" sign="positive" minvalue="1"/>
    <edge id="Wip1:p53_K:1" from="Wip1" to="p53_K" sign="negative" minvalue="1"/>
    <edge id="p53_A:p53_K:1" from="p53_A" to="p53_K" sign="negative" minvalue="1"/>
    <edge id="Sirt_1:p53_K:1" from="Sirt_1" to="p53_K" sign="negative" minvalue="1"/>
    <edge id="ATM:miR_34a:1" from="ATM" to="miR_34a" sign="positive" minvalue="1"/>
```

```
<edge id="p53:miR_34a:1" from="p53" to="miR_34a" sign="positive" minvalue="1"/>
<edge id="lnc_XIST:miR_34a:1" from="lnc_XIST" to="miR_34a" sign="negative" minvalue="1"/>
<edge id="ATM:miR_16:1" from="ATM" to="miR_16" sign="positive" minvalue="1"/>
<edge id="p53:miR_16:1" from="p53" to="miR_16" sign="positive" minvalue="1"/>
<edge id="lnc_XIST:miR_16:1" from="lnc_XIST" to="miR_16" sign="negative" minvalue="1"/>
<edge id="c_Myc:miR_16:1" from="c_Myc" to="miR_16" sign="negative" minvalue="1"/>
<edge id="HDAC1:miR_16:1" from="HDAC1" to="miR_16" sign="negative" minvalue="1"/>
<edge id="DNA_Damage:miR_449a:1" from="DNA_Damage" to="miR_449a" sign="positive" minvalue="1"/>
<edge id="lnc_XIST:miR_449a:1" from="lnc_XIST" to="miR_449a" sign="negative" minvalue="1"/>
<edge id="miR_34a:lnc_XIST:1" from="miR_34a" to="lnc_XIST" sign="negative" minvalue="1"/>
<edge id="miR_16:lnc_XIST:1" from="miR_16" to="lnc_XIST" sign="negative" minvalue="1"/>
<edge id="miR_449a:lnc_XIST:1" from="miR_449a" to="lnc_XIST" sign="negative" minvalue="1"/>
<edge id="YY1:lnc_XIST:1" from="YY1" to="lnc_XIST" sign="positive" minvalue="1"/>
<edge id="p53_A:p21:1" from="p53_A" to="p21" sign="positive" minvalue="1"/>
<edge id="lnc_XIST:p21:1" from="lnc_XIST" to="p21" sign="negative" minvalue="1"/>
<edge id="c_Myc:p21:1" from="c_Myc" to="p21" sign="negative" minvalue="1"/>
<edge id="YY1:p21:1" from="YY1" to="p21" sign="negative" minvalue="1"/>
<edge id="HDAC1:p21:1" from="HDAC1" to="p21" sign="negative" minvalue="1"/>
<edge id="BMI1:p21:1" from="BMI1" to="p21" sign="negative" minvalue="1"/>
<edge id="Caspase3:p21:1" from="Caspase3" to="p21" sign="negative" minvalue="1"/>
<edge id="p53_A:p53INP1:1" from="p53_A" to="p53INP1" sign="positive" minvalue="1"/>
<edge id="p53_K:p53INP1:1" from="p53_K" to="p53INP1" sign="positive" minvalue="1"/>
<edge id="miR_34a:c_Myc:1" from="miR_34a" to="c_Myc" sign="negative" minvalue="1"/>
<edge id="miR_449a:c_Myc:1" from="miR_449a" to="c_Myc" sign="negative" minvalue="1"/>
<edge id="p21:c_Myc:1" from="p21" to="c_Myc" sign="negative" minvalue="1"/>
<edge id="C_Met:c_Myc:1" from="C_Met" to="c_Myc" sign="positive" minvalue="1"/>
<edge id="RB:c_Myc:1" from="RB" to="c_Myc" sign="negative" minvalue="1"/>
<edge id="E2F1:c_Myc:1" from="E2F1" to="c_Myc" sign="positive" minvalue="1"/>
<edge id="miR_34a:YY1:1" from="miR_34a" to="YY1" sign="negative" minvalue="1"/>
<edge id="c_Myc:YY1:1" from="c_Myc" to="YY1" sign="positive" minvalue="1"/>
<edge id="RB:YY1:1" from="RB" to="YY1" sign="negative" minvalue="1"/>
<edge id="miR_34a:C_Met:1" from="miR_34a" to="C_Met" sign="negative" minvalue="1"/>
<edge id="miR_449a:C_Met:1" from="miR_449a" to="C_Met" sign="negative" minvalue="1"/>
<edge id="c_Myc:C_Met:1" from="c_Myc" to="C_Met" sign="positive" minvalue="1"/>
<edge id="ATM:Cdc25A:1" from="ATM" to="Cdc25A" sign="negative" minvalue="1"/>
<edge id="miR_34a:Cdc25A:1" from="miR_34a" to="Cdc25A" sign="negative" minvalue="1"/>
<edge id="miR_16:Cdc25A:1" from="miR_16" to="Cdc25A" sign="negative" minvalue="1"/>
<edge id="miR_449a:Cdc25A:1" from="miR_449a" to="Cdc25A" sign="negative" minvalue="1"/>
<edge id="miR_34a:CDK46_CycD:1" from="miR_34a" to="CDK46_CycD" sign="negative" minvalue="1"/>
<edge id="miR_16:CDK46_CycD:1" from="miR_16" to="CDK46_CycD" sign="negative" minvalue="1"/>
<edge id="miR_449a:CDK46_CycD:1" from="miR_449a" to="CDK46_CycD" sign="negative" minvalue="1"/>
<edge id="p21:CDK46_CycD:1" from="p21" to="CDK46_CycD" sign="negative" minvalue="1"/>
<edge id="Cdc25A:CDK46_CycD:1" from="Cdc25A" to="CDK46_CycD" sign="positive" minvalue="1"/>
<edge id="miR_34a:CDK2_CycE:1" from="miR_34a" to="CDK2_CycE" sign="negative" minvalue="1"/>
<edge id="miR_16:CDK2_CycE:1" from="miR_16" to="CDK2_CycE" sign="negative" minvalue="1"/>
<edge id="miR_449a:CDK2_CycE:1" from="miR_449a" to="CDK2_CycE" sign="negative" minvalue="1"/>
<edge id="p21:CDK2_CycE:1" from="p21" to="CDK2_CycE" sign="negative" minvalue="1"/>
<edge id="Cdc25A:CDK2_CycE:1" from="Cdc25A" to="CDK2_CycE" sign="positive" minvalue="1"/>
<edge id="E2F1:CDK2_CycE:1" from="E2F1" to="CDK2_CycE" sign="positive" minvalue="1"/>
<edge id="CDK46_CycD:RB:1" from="CDK46_CycD" to="RB" sign="negative" minvalue="1"/>
<edge id="CDK2_CycE:RB:1" from="CDK2_CycE" to="RB" sign="negative" minvalue="1"/>
<edge id="ATM:E2F1:1" from="ATM" to="E2F1" sign="positive" minvalue="1"/>
<edge id="miR_34a:E2F1:1" from="miR_34a" to="E2F1" sign="negative" minvalue="1"/>
```

```
437     <edge id="miR_449a:E2F1:1" from="miR_449a" to="E2F1" sign="negative" minvalue="1"/>
438     <edge id="c_Myc:E2F1:1" from="c_Myc" to="E2F1" sign="positive" minvalue="1"/>
439     <edge id="Cdc25A:E2F1:1" from="Cdc25A" to="E2F1" sign="positive" minvalue="1"/>
440     <edge id="RB:E2F1:1" from="RB" to="E2F1" sign="negative" minvalue="1"/>
441     <edge id="Sirt_1:E2F1:1" from="Sirt_1" to="E2F1" sign="negative" minvalue="1"/>
442     <edge id="miR_34a:Sirt_1:1" from="miR_34a" to="Sirt_1" sign="negative" minvalue="1"/>
443     <edge id="miR_449a:Sirt_1:1" from="miR_449a" to="Sirt_1" sign="negative" minvalue="1"/>
444     <edge id="E2F1:Sirt_1:1" from="E2F1" to="Sirt_1" sign="positive" minvalue="1"/>
445     <edge id="HDAC1:Sirt_1:1" from="HDAC1" to="Sirt_1" sign="negative" minvalue="1"/>
446     <edge id="DNA_Damage:HDAC1:1" from="DNA_Damage" to="HDAC1" sign="negative" minvalue="1"/>
447     <edge id="miR_34a:HDAC1:1" from="miR_34a" to="HDAC1" sign="negative" minvalue="1"/>
448     <edge id="miR_449a:HDAC1:1" from="miR_449a" to="HDAC1" sign="negative" minvalue="1"/>
449     <edge id="Sirt_1:HDAC1:1" from="Sirt_1" to="HDAC1" sign="negative" minvalue="1"/>
450     <edge id="miR_16:BMI1:1" from="miR_16" to="BMI1" sign="negative" minvalue="1"/>
451     <edge id="c_Myc:BMI1:1" from="c_Myc" to="BMI1" sign="positive" minvalue="1"/>
452     <edge id="E2F1:BMI1:1" from="E2F1" to="BMI1" sign="positive" minvalue="1"/>
453     <edge id="p53_K:PUMA:1" from="p53_K" to="PUMA" sign="positive" minvalue="1"/>
454     <edge id="miR_34a:BCL2:1" from="miR_34a" to="BCL2" sign="negative" minvalue="1"/>
455     <edge id="miR_16:BCL2:1" from="miR_16" to="BCL2" sign="negative" minvalue="1"/>
456     <edge id="miR_449a:BCL2:1" from="miR_449a" to="BCL2" sign="negative" minvalue="1"/>
457     <edge id="PUMA:BCL2:1" from="PUMA" to="BCL2" sign="negative" minvalue="1"/>
458     <edge id="p53_K:BAX:1" from="p53_K" to="BAX" sign="positive" minvalue="1"/>
459     <edge id="BCL2:BAX:1" from="BCL2" to="BAX" sign="negative" minvalue="1"/>
460     <edge id="p21:Caspase3:1" from="p21" to="Caspase3" sign="negative" minvalue="1"/>
461     <edge id="BCL2:Caspase3:1" from="BCL2" to="Caspase3" sign="negative" minvalue="1"/>
462     <edge id="BAX:Caspase3:1" from="BAX" to="Caspase3" sign="positive" minvalue="1"/>
463     <edge id="p53:Poliferation:1" from="p53" to="Poliferation" sign="negative" minvalue="1"/>
464     <edge id="CDK2_CycE:Poliferation:1" from="CDK2_CycE" to="Poliferation" sign="positive"
            minvalue="1"/>
465     <edge id="p21:Senescence:1" from="p21" to="Senescence" sign="positive" minvalue="1"/>
466     <edge id="CDK2_CycE:Senescence:1" from="CDK2_CycE" to="Senescence" sign="negative"
            minvalue="1"/>
467     <edge id="Caspase3:Apoptosis:1" from="Caspase3" to="Apoptosis" sign="positive" minvalue="1
            "/>
468   </graph>
469 </gxl>
```
