# Supplementary material for: A Boolean Model of the Proliferative Role of the lncRNA XIST in Non-Small Cell Lung Cancer Cells
Source: Biology (Basel). 2022 Mar 22;11(4):480. doi: 10.3390/biology11040480 (PMC9024590; doi:10.3390/biology11040480)
Supplement: Supplementary file 1 [file biology-11-00480-s001.zip › Figure S1.pdf]

A Boolean model of the proliferative role of the lncRNA XIST in non-small cell lung cancer cells

S1 Figure

Shantanu Gupta[1, +, *], Daner A. Silveira[2,+], Ronaldo F. Hashimoto[1, +], and Jose Carlos M. Mombach[2,+,*]

[1]Instituto de Matemática e Estatística, Departamento de Ciência da Computação, Universidade de São Paulo, Rua do Matão 1010, 05508-090, São Paulo - SP, Brasil

[2]Departamento de Física, Universidade Federal de Santa Maria, RS, Brazil

[*]Corresponding authors: shantanu.gupta@ime.usp.br ; jcmombach@ufsm.br

[+]These authors contributed equally to this work

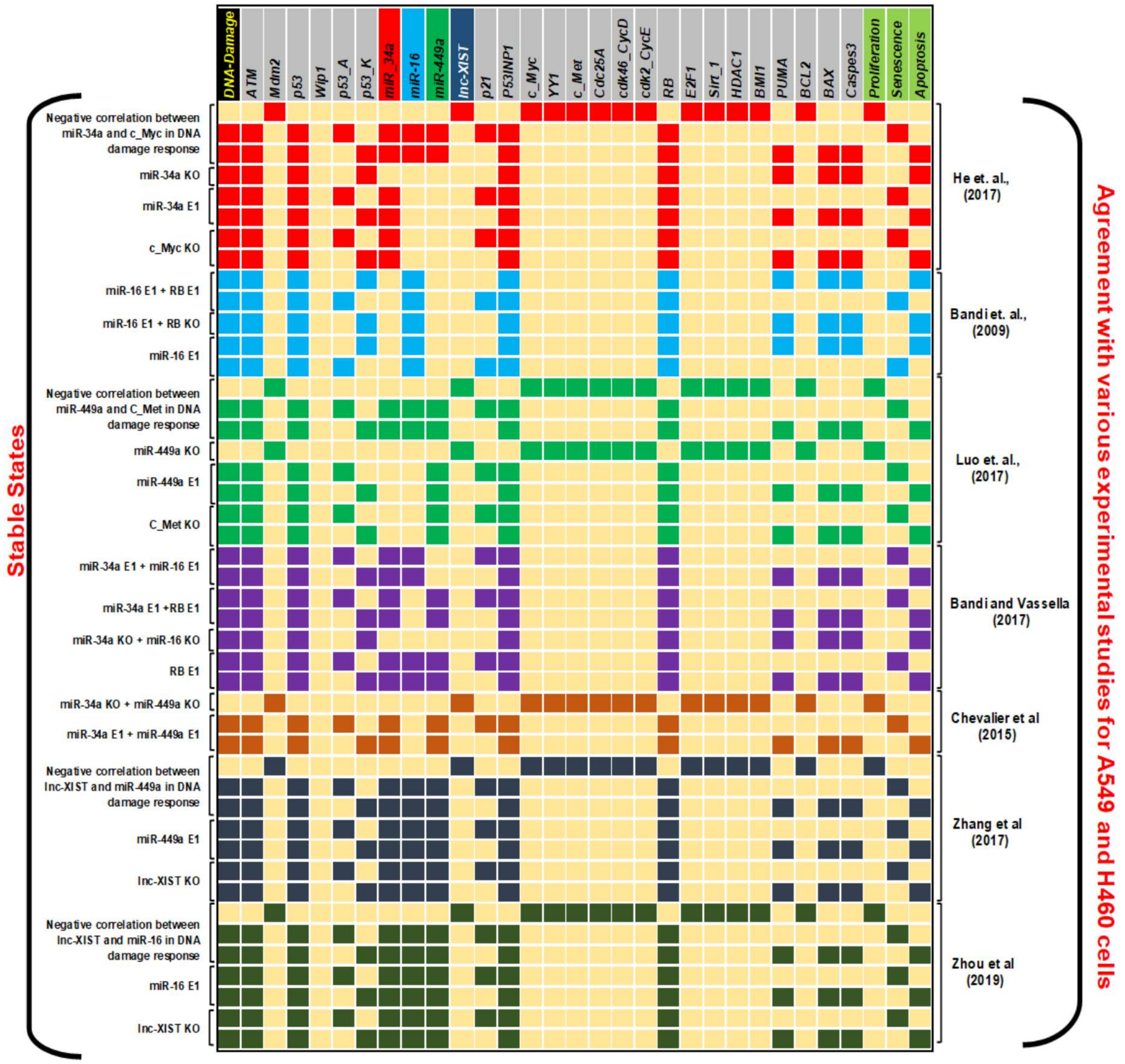

Figure S1. Referential experimental studies used to develop the network (see Table. 1 in main article). E1 represents gain of function (GoF) and KO stands for LoF of the corresponding molecules. Yellow cells denote inactivation (OFF), whereas colored cells such as red, blue, green, violet, brown, black and dark green denote activation (ON). Each line represents a stable state. The left-hand side presents perturbations, whereas the right-hand side its corresponding study. Red cells correspond to He et. al. study [1]. Blue cells: Bandi et. al. [2]. Green cells: Luo et al study [3]. Violet cells: Bandi and vassella et al [4]. Brown cells: Chevalier et al study [5]. Black cells: Zhang et al [6]. Dark green: Zhou et al [7].

References

[1] X. He, A. Yang, D.G. McDonald, E.C. Riemer, K.N. Vanek, B.A. Schulte, G.Y. Wang, MiR-34a modulates ionizing radiation-induced senescence in lung cancer cells, Oncotarget. 8 (2017) 69797.

[2] N. Bandi, S. Zbinden, M. Gugger, M. Arnold, V. Kocher, L. Hasan, A. Kappeler, T. Brunner, E. Vassella, miR-15a and miR-16 Are Implicated in Cell Cycle Regulation in a Rb-Dependent Manner and Are Frequently Deleted or Down-regulated in Non–Small Cell Lung Cancer, Cancer Res. 69 (2009) 5553–5559. https://doi.org/10.1158/0008-5472.CAN-08-4277.

[3] W. Luo, B. Huang, Z. Li, H. Li, L. Sun, Q. Zhang, X. Qiu, E. Wang, MicroRNA-449a is downregulated in non-small cell lung cancer and inhibits migration and invasion by targeting c-Met, PloS One. 8 (2013) e64759.

[4] N. Bandi, E. Vassella, miR-34a and miR-15a/16 are co-regulated in non-small cell lung cancer and control cell cycle progression in a synergistic and Rb-dependent manner, Molecular Cancer. 10 (2011) 55.

[5] B. Chevalier, A. Adamiok, O. Mercey, D.R. Revinski, L.-E. Zaragosi, A. Pasini, L. Kodjabachian, P. Barbry, B. Marcet, miR-34/449 control apical actin network formation during multiciliogenesis through small GTPase pathways, Nature Communications. 6 (2015) 8386. https://doi.org/10.1038/ncomms9386.

[6] Y. Zhang, X. Li, Y. Hou, N. Fang, J. You, Q. Zhou, The lncRNA XIST exhibits oncogenic properties via regulation of miR-449a and Bcl-2 in human non-small cell lung cancer, Acta Pharmacologica Sinica. 38 (2017) 371–381. https://doi.org/10.1038/aps.2016.133.

[7] X. Zhou, X. Xu, C. Gao, Y. Cui, XIST promote the proliferation and migration of non-small cell lung cancer cells via sponging miR-16 and regulating CDK8 expression, Am J Transl Res. 11 (2019) 6196–6206.
